# Portable solutions for plant pathogen diagnostics: development, usage, and future potential

**DOI:** 10.3389/fmicb.2025.1516723

**Published:** 2025-01-31

**Authors:** Anurag Yadav, Kusum Yadav

**Affiliations:** ^1^Department of Microbiology, C. P. College of Agriculture, Sardarkrushinagar Dantiwada Agricultural University, Banaskantha, India; ^2^Department of Biochemistry, University of Lucknow, Lucknow, India

**Keywords:** portable diagnostics, point-of-care testing, plant pathogens, portable biosensors, lab-on-a-chip

## Abstract

The increasing prevalence of plant pathogens presents a critical challenge to global food security and agricultural sustainability. While accurate, traditional diagnostic methods are often time-consuming, resource-intensive, and unsuitable for real-time field applications. The emergence of portable diagnostic tools represents a paradigm shift in plant disease management, offering rapid, on-site detection of pathogens with high accuracy and minimal technical expertise. This review explores portable diagnostic technologies’ development, deployment, and future potential, including handheld analyzers, smartphone-integrated systems, microfluidics, and lab-on-a-chip platforms. We examine the core technologies underlying these devices, such as biosensors, nucleic acid amplification techniques, and immunoassays, highlighting their applicability to detect bacterial, viral, and fungal pathogens in diverse agricultural settings. Furthermore, the integration of these devices with digital technologies, including the Internet of Things (IoT), artificial intelligence (AI), and machine learning (ML), is transforming disease surveillance and management. While portable diagnostics have clear advantages in speed, cost-effectiveness, and user accessibility, challenges related to sensitivity, durability, and regulatory standards remain. Innovations in nanotechnology, multiplex detection platforms, and personalized agriculture promise to further enhance the efficacy of portable diagnostics. By providing a comprehensive overview of current technologies and exploring future directions, this review underscores the critical role of portable diagnostics in advancing precision agriculture and mitigating the impact of plant pathogens on global food production.

## 1 Introduction

Rapid and accurate diagnosis of plant pathogens is a cornerstone in ensuring global food security, safeguarding ecosystem integrity, and sustaining agricultural productivity. As plant diseases increasingly challenge crop yields and quality, the timely detection and identification of bacterial, viral, fungal, and other phytopathogens have become paramount in guiding efficient disease management and limiting the economic losses associated with epidemics (Savary and Willocquet, 2020; Ali et al., 2021). Traditional laboratory-based diagnostic methodologies—such as plating, microscopy, enzyme-linked immunosorbent assays (ELISAs), and polymerase chain reaction (PCR)—have undoubtedly advanced our capacity to detect pathogens at remarkable levels of accuracy and sensitivity (Boonham et al., 2014). However, these approaches often require specialized equipment, stable infrastructure, and trained personnel, creating practical bottlenecks in real-world, resource-limited scenarios.

Portable diagnostic technologies—ranging from handheld biosensors and smartphone-based platforms to lab-on-a-chip devices—are promising tools that bring the diagnostic power of advanced molecular assays directly into the field (Srinivasan and Tung, 2015). Such innovations align well with the broader paradigm shift toward precision agriculture, enabling evidence-based decision-making, early warning systems, and improved surveillance networks that integrate seamlessly into the Internet of Things (IoT) and cloud-based architectures (Srinivasan and Tung, 2015).

Despite this momentum, notable gaps remain. While several reviews discuss molecular methods and high-throughput genomics-based diagnostics (Studholme et al., 2011; Aslam et al., 2017; Hariharan and Prasannath, 2021; Ijaz et al., 2022; Chakraborty and Chakraborty, 2021), a comprehensive synthesis focusing explicitly on the evolving landscape of portable diagnostic platforms and their integration into digital agronomy frameworks has yet to be presented. Existing literature often addresses individual technologies—such as loop-mediated isothermal amplification (LAMP) devices or smartphone-based lateral flow assays—in isolation. However, a holistic review that connects these diverse technologies, elucidates their underlying principles, and critically examines their field applicability, data integration strategies, and regulatory frameworks is lacking. Moreover, as portable diagnostics move from proof-of-concept prototypes toward scalable, commercialized products, there is a pressing need to map out the challenges in validation, standardization, and quality assurance that must be surmounted for widespread adoption (Ahmad-Nejad et al., 2021). A recent review article by Nguyen et al. (2024) focuses on biosensors as one specific technology for plant disease diagnosis and highlights advances in biosensor technologies, focusing on technical and functional innovations in their design and application. Another review article provides a comprehensive overview of plant pathogen detection techniques, encompassing cultivation, PCR, sequencing-based methods, and immunoassays. It includes portable biosensors as a subsection, highlighting recent advancements and their application in point-of-care diagnostics (Venbrux et al., 2023). The above two articles provide a holistic, multidisciplinary perspective on portable plant pathogen diagnostics, while broader scope, deeper integration of digital technologies, and consideration of real-world challenges and future innovations set the present review apart as a more comprehensive resource.

This review addresses critical knowledge gaps in plant pathogen diagnostics by offering a comprehensive perspective on portable solutions. It examines the core principles and technologies behind handheld analyzers, smartphone-integrated biosensors, and microfluidic-based lab-on-a-chip systems while highlighting the transformative role of IoT, cloud-based analytics, artificial intelligence (AI), and machine learning (ML) in enhancing data interpretation, connectivity, and global disease surveillance.

The review explores applications across bacterial, viral, and fungal pathogens, emphasizing how on-site testing informs disease surveillance, management decisions, and early warning systems. It addresses performance evaluation, validation in resource-limited settings, regulatory challenges, and commercial applicability to bridge the gap between innovation and real-world implementation. Additionally, it identifies future innovations, such as scalable manufacturing, cost reduction, and stakeholder engagement, aimed at advancing accessible and effective portable diagnostics. Through this integrated approach, the review provides actionable insights to drive advancements in plant pathogen diagnostics.

By providing a comprehensive analysis of the state-of-the-art in portable phytopathogen diagnostic technologies—alongside insights into data management, standardization, and integrated decision-making—this review will empower researchers, policymakers, industry stakeholders, and practitioners. Such an integrative understanding will not only help advance this rapidly evolving field but also support evidence-driven disease management strategies, enhance global surveillance networks, and pave the way for more resilient and sustainable agricultural systems.

## 2 Portable diagnostic technologies for plant pathogen detection

### 2.1 General principles and core components of phytopathogen detection devices

Portable phytopathogen detection devices integrate actuators and sensors initially developed for consumer electronics, including smartphones and smartwatches, to enable on-site, real-time diagnostics. Actuator components, such as miniaturized light-emitting diodes (LEDs), are capable of emitting wavelengths spanning the visible range (approximately 400–700 nm) and have been employed to stimulate fluorescence or other optical responses in biochemical assays, particularly in the detection of pathogen-associated molecular markers. LEDs for portable biosensing, when coupled with smartphone imaging, have been well documented, offering a promising route toward rapid, field-based diagnostics of plant pathogens (Gudkov et al., 2023). Similarly, near-field communication (NFC) modules operating at 13.56 MHz allow short-range interactions with magnetic or resistive sensing elements, thereby enabling wireless activation of biosensors and facilitating assessments of soil moisture or other environmental parameters that influence pathogen spread (Gawade, 2022).

Display screens in smartphones and smartwatches, with resolutions often exceeding 720 × 1,280 px, can emit controlled wavelength outputs—such as red (628 nm), green (536 nm), and blue (453 nm)—to serve as dynamic light sources for colorimetric analyses of plant extracts. In addition, vibration motors providing haptic feedback in the range of 130–180 Hz can be leveraged to enhance assay kinetics by mixing reagents directly in the field, while integrated speakers emitting acoustic signals are being explored to disrupt sample matrices or stimulate particular biochemical reactions (Fan et al., 2021). The integration of thermal actuators is also critical, as these elements enable precise temperature control essential for nucleic acid amplification tests (NATs), including polymerase chain reaction (PCR) assays, thus facilitating on-the-spot genomic detection of pathogens without the need for a fully equipped laboratory.

Sensor components are equally transformative. Imaging detectors, including high-resolution smartphone cameras equipped for UV–Vis spectrometry, provide powerful means to capture fluorescence, color changes, or other optical indicators of infection. These imaging strategies have proven helpful for plant disease phenotyping and early stress detection (Zubler and Yoon, 2020). Environmental light sensors enhance sensitivity by accounting for ambient conditions and improving the reliability of fluorescence or colorimetric quantifications. NFC readers serve dual roles as communication modules and near-field sensors, enabling rapid, localized measurements vital for wearable and in-field biosensors.

Initially designed for human-device interaction, capacitive touchscreen sensors are increasingly recognized for their ability to detect subtle changes in pressure, moisture, or conductivity when brought into contact with plant tissues, thereby providing indirect indications of infection or physiological stress. Wearable photodetectors integrated into smartwatches further expand the scope for continuous, mobile monitoring of plant health markers, and microphones sensitive to acoustic signals emitted during microbial metabolism or pathogen-vector interactions offer unique, non-invasive diagnostic approaches (Xu et al., 2024). Coupling these sensory capabilities with geolocation data from GPS modules enables spatial mapping of disease outbreaks and supports the implementation of data-driven management strategies (Xin et al., 2009).

The convergence of these actuators and sensors within portable platforms is revolutionizing how researchers and field professionals monitor phytopathogens. Smartphones and smartwatches have evolved into versatile biosensing tools, providing real-time imaging, thermal regulation, optical stimulation, wireless connectivity, and acoustic or tactile feedback (Buja et al., 2021). These devices facilitate on-site nucleic acid amplification and rapid pathogen detection and enable continuous environmental and plant health monitoring. These integrated technologies can guide timely interventions by tracking chlorophyll fluorescence fluctuations indicative of early disease onset, mapping pathogen prevalence across agricultural landscapes, and detecting subtle changes in leaf temperature profiles. As a result, mobile devices equipped with tailored actuators and sensors are poised to become indispensable in modern precision agriculture, improving our capacity to detect, map, and manage phytopathogens and safeguarding global crop productivity and food security.

**Figure 1:**
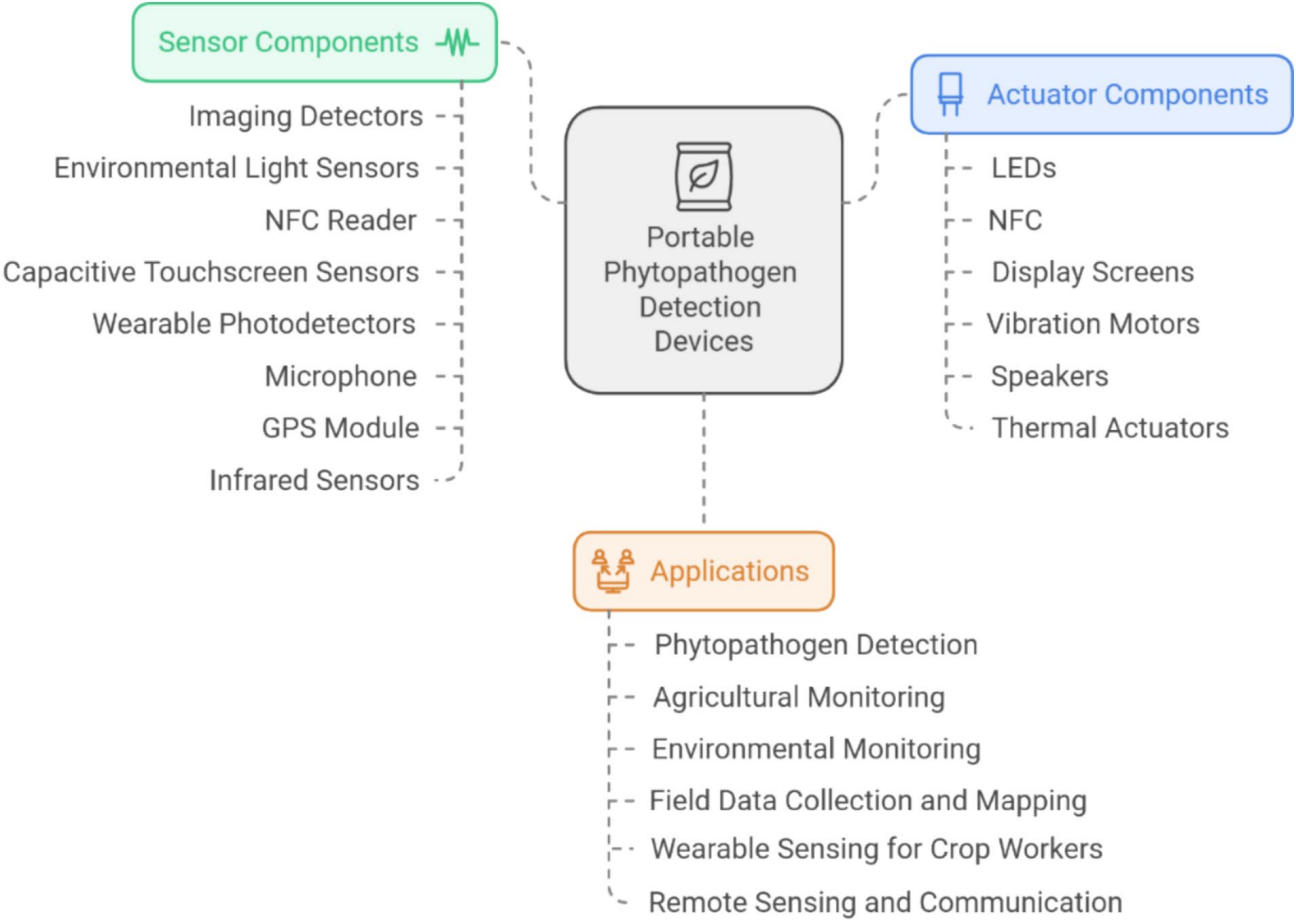
Components of portable phytopathogen detection devices and their applications.

Figure 1 shows that portable phytopathogen detection devices integrate sensor components (such as imaging detectors, light sensors, and GPS modules) and actuator components (like LEDs and thermal actuators) to enable various applications, including phytopathogen detection, agricultural monitoring, and remote sensing for enhanced crop management.

### 2.2 Classification and overview

**Figure 2:**
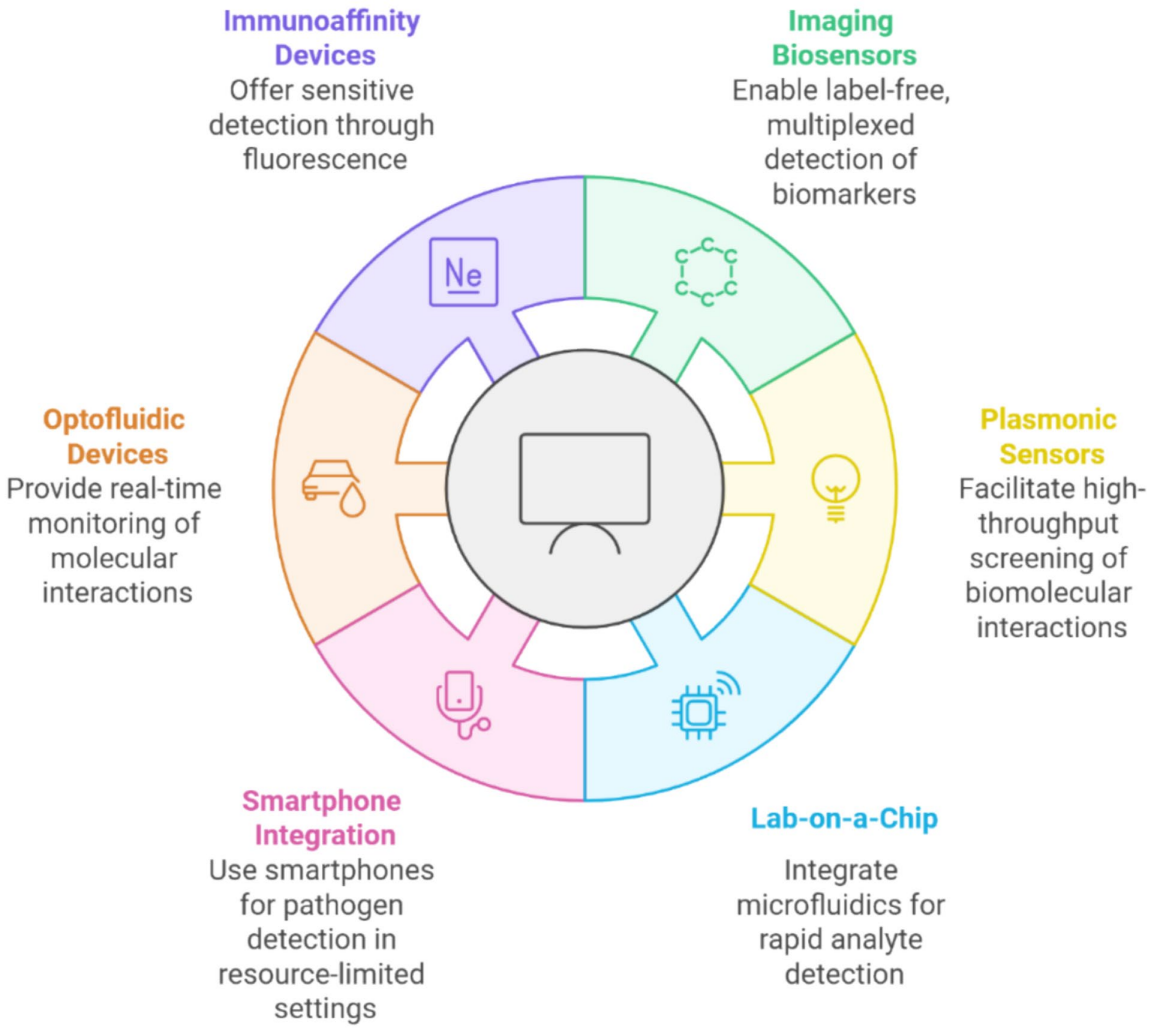
Emerging technologies in biosensing for rapid and sensitive detection.

Figure 2 illustrates various advanced biosensing technologies categorized based on their unique functionalities. Immunoaffinity devices provide sensitive detection using fluorescence techniques, while imaging biosensors enable label-free, multiplexed biomarker detection. Plasmonic sensors facilitate high-throughput screening of biomolecular interactions, and lab-on-a-chip systems integrate microfluidics for rapid analyte detection. Additionally, optofluidic devices offer real-time monitoring of molecular interactions, and smartphone integration enables biosensing in resource-limited settings by leveraging portable and accessible technologies. Together, these innovations highlight the convergence of biosensing technologies’ precision, portability, and scalability.

#### 2.2.1 Handheld analyzers and mobile biosensors

Handheld analyzers and mobile biosensors are essential for rapid, on-site diagnostics, especially in environments where traditional laboratory infrastructure is unavailable. These portable devices integrate advanced biosensing technologies, enabling the detection of pathogens, chemicals, and other analytes directly in the field. Their compact size, ease of use, and fast response times make them highly suited for clinical diagnostics, environmental monitoring, and resource-limited settings that require immediate results.

A significant innovation in this space is using plasmonic biosensors, which utilize nanohole arrays and CMOS camera technology for sensitive, label-free viral detection. Its adaptability lies in using a plasmonic chip surface coated with specific antibodies, which can be modified to target different viral types depending on the surface coating (Cetin et al., 2021). Similarly, portable electrochemical biosensors, like those used for detecting hepatitis C, offer Bluetooth-enabled real-time monitoring. These devices use cyclic voltammetry to identify viral markers and boast a wireless setup with over three hours of battery life, making them ideal for mobile diagnostics (de Campos da Costa et al., 2019).

Integrating smartphones into biosensing technologies further enhances these tools by providing advanced data processing, storage, and sharing capabilities. For instance, a “three-in-one” biosensor detects infections and provides diagnostic information for each stage of the infection cycle, enabling continuous patient health monitoring (Dou et al., 2022).

Moreover, microfluidic technology has been incorporated into portable analyzers, allowing rapid and multiplexed detection. A paper-based microfluidic biosensor, for instance, has been developed to detect viral pathogens in samples. This electrochemical immunosensing platform can simultaneously perform enzyme-linked immunosorbent assays (ELISA) on multiple samples, offering a low-cost and user-friendly solution for point-of-care diagnostics (Zhao and Liu, 2016).

Another valuable addition to mobile diagnostic technology is interferometric biosensors, such as the Young interferometer sensor. These highly sensitive devices combine optical sensors with antibody-antigen recognition for rapid and accurate viral detection. Their portability and ease of use make them especially useful in remote areas that lack sophisticated laboratory facilities (Ymeti et al., 2007).

**Figure 3:**
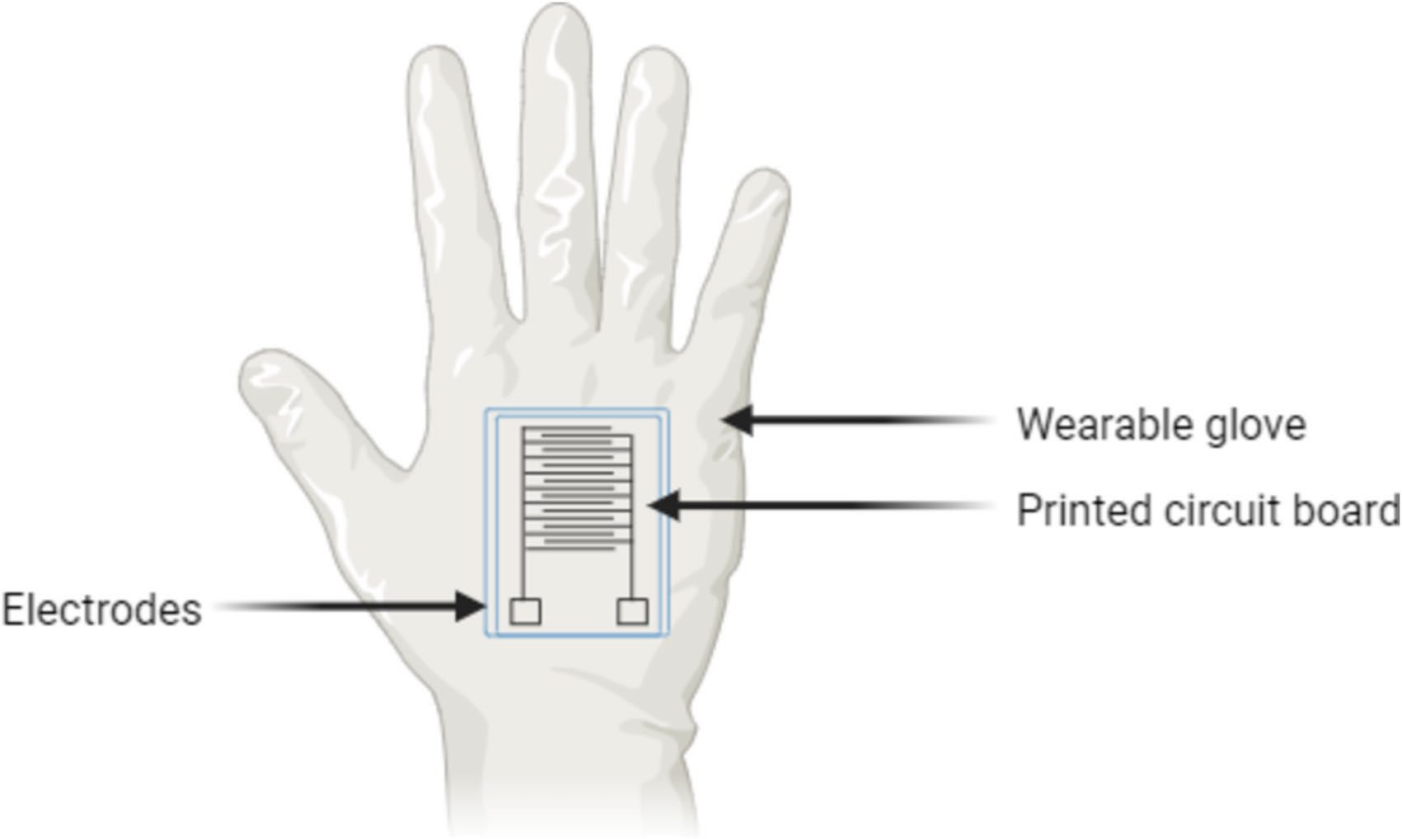
Wearable biosensor glove with integrated circuit for on-site pathogen detection.

Beyond medical applications, handheld analyzers have also proven valuable in agricultural settings. For example, a bioelectric recognition assay has been tested to detect plant pathogens like Potato virus Y and Cucumber mosaic virus. This technology can process up to 96 samples in approximately 70 min, making it ideal for on-site pathogen diagnostics, where quick results are critical for preventing widespread crop damage (Perdikaris et al., 2011). Another example of this advancement is depicted in Figure 3, which demonstrates the design of a wearable biosensor glove with an integrated printed circuit board for real-time pathogen detection in various field conditions.

#### 2.2.2 Smartphone and smartwatch integrated diagnostic tools

Smartphone and smartwatch integrated diagnostic tools are transforming phytopathogen detection by combining molecular assays, AI-powered image analysis, and sensor technologies, providing rapid, accurate, and real-time pathogen identification in agricultural settings. Tools such as Smart LAMP use Loop-Mediated Isothermal Amplification (LAMP) to detect pathogens like *Ralstonia solanacearum* in crops like tomatoes and potatoes. This setup eliminates the need for complex thermocyclers operating at a single temperature. Using the smartphone camera to capture color changes in the LAMP reaction, the tool provides visual indicators of infection in real-time, making it highly accessible for remote or resource-limited environments (Ivanov et al., 2021).

The Biomeme two3 device integrates qPCR with smartphone connectivity, enabling DNA and RNA analysis for pathogens such as *Fusarium oxysporum* in bananas and tomatoes. This mobile platform uses fluorescence signals to quantify pathogen load in approximately 45 min, displaying results via a smartphone app. Smartwatches can extend the utility of this platform by receiving and displaying diagnostic alerts, allowing for immediate field-level responses even when the smartphone is not in use. This real-time data can be shared remotely, making it ideal for rapid, informed decision-making (Buja et al., 2021). Similarly, nano-sensor technology uses VOC detection to analyze biomarkers emitted by infected plants, allowing early disease detection even before visible symptoms appear. This smartphone-based system has proven effective in crops like wheat and potatoes, helping farmers proactively manage crop health (Li et al., 2019).

For image-based detection, ViT-SmartAgri utilizes a Vision Transformer model to classify tomato leaf images, distinguishing between healthy and diseased plants with high accuracy. By capturing and processing leaf images through the smartphone camera, ViT-SmartAgri provides instant diagnostic feedback, supporting early intervention against diseases like late blight and mosaic virus. Smartwatches can act as companion devices, offering quick access to diagnostic summaries and alerts, further improving efficiency in field operations (Alzahrani and Alsaade, 2023).

The Agdia ImmunoStrip^®^ combines lateral flow assays with smartphone technology to detect *Candidatus Liberibacter asiaticus*, the pathogen behind the citrus greening disease. After the DNA amplification process, the test line’s visibility on the strip provides a straightforward readout, which can be captured and analyzed by a smartphone app. This tool has been instrumental in Florida citrus groves, allowing quick, in-field diagnostic results to mitigate disease spread (Russell et al., 2015).

#### 2.2.3 Microfluidics and lab-on-a-chip systems

Portable microfluidic and lab-on-a-chip (LOC) systems fundamentally transform phytopathogen detection in agriculture. These compact devices integrate various functions, including sample preparation, amplification, and detection, onto a single chip, enabling quick responses to pathogen outbreaks and supporting proactive crop disease management. Examples such as the CRISPR-Cas13a-based centrifugal microfluidic system exemplifies these advancements, automating processes like nucleic acid extraction and detection within a single device, achieving sensitivity levels as low as one copy per reaction in plasmid samples, which is particularly useful for field applications requiring high sensitivity (Zhang et al., 2024).

Similarly, hand-powered microfluidic devices enable affordable sample processing by utilizing simple mechanisms, such as syringes, to facilitate digital PCR applications without external power sources, making them ideal for remote agricultural settings where cost-effective, high-throughput diagnostics are essential (Xie et al., 2021). Other LOC systems use LAMP-based technologies with electrochemical sensors capable of operating at lower temperatures (39°C), delivering real-time results within 20 min—an advantage for rapid, in-field diagnostics of viral phytopathogens (Hsieh et al., 2015).

**Figure 4:**
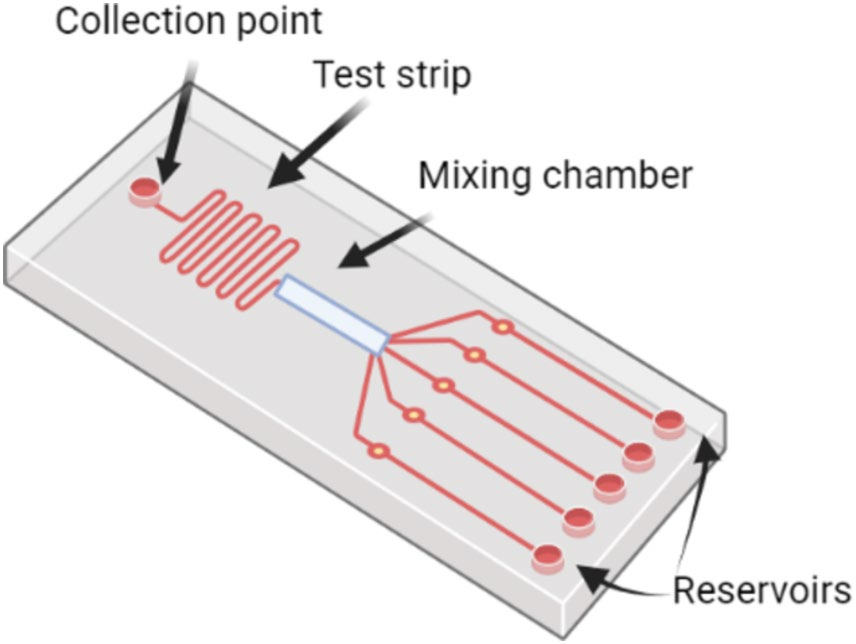
Schematic of a microfluidic lab-on-a-chip system for pathogen detection.

Figure 4 illustrates a typical microfluidic LOC system for pathogen detection. The chip consists of several key components, including a collection point where the sample is introduced, a test strip for initial sample processing, and a mixing chamber that allows the sample to interact with reagents. The chip is also equipped with multiple reservoirs, which store necessary reagents and direct them through the system for diagnostic processes. This configuration enables efficient, automated pathogen detection by minimizing manual handling, integrating various analytical methods, and facilitating high-throughput testing suitable for on-field diagnostics.

More advanced microfluidic devices, such as the mChip, expand on these capabilities with fluorescence-based detection for pathogens like *Xylella fastidiosa*, impacting crops such as olives. When integrated with smartphone-based fluorescence readers, this platform enables early pathogen detection with high accuracy, helping farmers in regions like Southern Italy promptly identify infections (Milson and Kevin, 2023). Similarly, multiplex platforms like PlexBio^™^ OptoSelect^™^ allow simultaneous detection of multiple pathogens. Using color-coded microbeads, PlexBio has successfully identified bacterial diseases such as spot, blight, and mosaic virus in Californian tomato farms within an hour, streamlining large-scale agricultural testing (Foudeh et al., 2012).

For simpler applications, paper-based microfluidic devices (e.g., μPADs) use capillary action for the colorimetric detection of pathogens without requiring external power. Such devices are precious in resource-limited environments across Southeast Asia, where visible color changes in response to pathogen presence enable users to interpret them more straightforwardly (Chen et al., 2021). Furthermore, ChemBio^™^ offers a chemiluminescent immunoassay-based solution that has proven effective in detecting pathogens associated with citrus greening disease in Chinese citrus groves, thereby allowing for tailored disease management practices (Hu et al., 2017).

Lastly, platforms like LabDisk use centrifugal forces for automated sample handling, directing fluids through preloaded reagents to reaction chambers for pathogen detection. This technology has been employed in vineyards to monitor *Botrytis cinerea*, responsible for grey mold, by reducing manual handling and facilitating high-throughput diagnostics suitable for large-scale field applications (Neethirajan et al., 2011).

### 2.3 Core technologies and methods employed in handheld analyzers

Portable point-of-care (POC) devices for phytopathogen detection are essential in agricultural and environmental management, enabling on-site diagnostics and immediate decision-making to curb plant diseases. These devices’ portability and user-friendliness are designed for field deployment, allowing use by individuals without specialized training. Lightweight and compact, POC devices are optimized for rapid, on-site testing, crucial in areas lacking lab access. Their ability to deliver quick results in a few minutes to hours compared to lab tests that may take days—supports timely intervention to prevent further pathogen spread, which is especially critical in large-scale agricultural settings where delays can lead to economic losses (Buja et al., 2021; Safavieh et al., 2014). Table 1 lists the core technologies used in portable diagnostic devices.

**Table 1:** Core technologies in portable diagnostic devices.

Core technology	Specific methods	Principles	Examples of use	References
Biosensors	Electrochemical, optical, plasmonic sensors	Detects specific pathogens by measuring biochemical reactions via electrical signals or light changes	Detection of bacterial pathogens like *Xylella fastidiosa* and viral pathogens such as Potato virus Y	Gutiérrez-Aguirre et al. (2014)
Nucleic acid amplification	PCR, LAMP, RPA	Amplifies pathogen DNA/RNA for detection without complex laboratory setups; operates under isothermal conditions	Field diagnostics for viruses such as Tomato Yellow Leaf Curl Virus in infected crops	Harper et al. (2010)
Immunoassays	ELISA, lateral flow assays	Uses antibodies to bind to pathogen antigens, producing a measurable signal for rapid pathogen detection	On-site detection of pathogens like *Phytophthora infestans* through colorimetric or fluorescence signals	Bergwerff and Van Knapen (2006)

The latest developments integrate nucleic acid amplification and optical detection, allowing these devices to differentiate pathogenic from non-pathogenic organisms—critical for reliable diagnostics (Hsieh et al., 2015). Durability is equally important, as these devices often endure harsh conditions. Features like ruggedized casings, moisture resistance, and self-contained power sources ensure reliability and minimize maintenance in varied agricultural environments (Kou et al., 2020).

#### 2.3.1 Biosensor technologies

Portable biosensor devices have revolutionized phytopathogen detection, enabling rapid, accurate, field-ready diagnostics supporting effective agricultural disease management. Key innovations include smartphone-based systems, LOC technology, bioluminescent cell-based sensors, and plasmonic biosensors, all offering practical alternatives to traditional laboratory methods.

Smartphone-based biosensors utilize smartphones’ cameras and processing power, often relying on colorimetric assays that change color upon pathogen detection. Custom apps then analyze these changes, providing rapid results ideal for resource-limited environments (Huang et al., 2018). LOC devices also enhance portability by miniaturizing lab processes onto a chip, allowing quick diagnostics with minimal reagents. For instance, LOC microcantilever-based biosensors can detect *Salmonella* at low concentrations of 10^3^ CFU^−1^ mL, demonstrating their efficacy in field applications (Ricciardi et al., 2010).

Bioluminescent cell-based biosensors employ genetically engineered cells that emit light in response to specific pathogens, enabling high-intensity nanomolar detection levels suitable for direct field diagnostics (Roda et al., 2011). Enzyme-based biosensors with metal-organic frameworks (MOFs) are another portable option; these smartphone-assisted sensors provide real-time colorimetric detection, making them a cost-effective choice for agricultural pathogen monitoring (Kou et al., 2020). Additionally, nanoparticle-based biosensors enable sensitive pathogen detection by leveraging nanoparticles’ high surface area and functional versatility. Gold nanoparticles (AuNPs), known for their optical properties, produce visible color changes that assist in detecting agrichemicals, such as pesticides, within seconds, which is ideal for on-site testing in resource-limited settings (Baek et al., 2017). Magnetic nanoparticles further support pathogen detection; biosensors using Fe_3_O_4_ nanoparticles with acetylcholinesterase are highly sensitive and reusable, well-suited for repeated agricultural tests (Chauhan and Pundir, 2011).

Plasmonic biosensors amplify optical signals using metal nanoparticles, facilitating phytopathogen detection. Handheld devices with plasmonic microarrays provide quantitative data through computational imaging, offering reliable results with minimal sample prep (Cetin et al., 2014). Plant-wearable biosensors employing gold nanoparticles monitor pesticides in real-time, transmitting data to a smartphone for precision application and supporting sustainable agricultural practices (Zhao et al., 2020). Moreover, mobile apps like LAMP-CAM enhance diagnostics by utilizing colorimetric detection to identify pathogens such as *Phytophthora infestans*, a primary cause of late blight in crops. LAMP-CAM interprets color changes detected by a smartphone camera and translates them into diagnostic results, improving accuracy in remote areas (Raguraman et al., 2021).

Electrochemical biosensors are also noteworthy. For example, apps like PSoC Programmer and PSoC Creator connect to electrochemical sensors via USB OTG or Bluetooth, detecting pathogens like *Xanthomonas oryzae* in rice and enabling real-time data visualization (Currie, 2021). Meanwhile, ViT-SmartAgri uses deep learning for plant disease identification, employing a Vision Transformer (ViT) model to accurately differentiate between healthy and diseased tomato plants, providing a user-friendly disease management solution (Barman et al., 2024). Cloud-connected portable LAMP systems enhance biosensor capabilities by enabling rapid, on-site detection of *Candidatus Liberibacter asiaticus*, the citrus greening pathogen. These devices integrate fluorescence-based detection with smartphone apps for real-time data sharing, aiding swift management decisions (Fujikawa & Iwanami, 2012). Data is transmitted to cloud services, supporting centralized analysis and rapid outbreak response. Table 2 describes the fabrication methods used to develop sensors in phytopathogen diagnostics.

**Table 2:** Fabrication techniques for sensors in phytopathogen diagnostics.

Sensor type	Fabrication methods	Key advantages	Target phytopathogens	References
Paper-based microfluidic sensors	Hydrophobization of paper to create microfluidic channels Inkjet printing of biomolecules for sensing zones	Simplified fabrication with scalability using commercial printing technologies	General phytopathogen diagnostics	Li et al. (2010)
Semiconductor-based biosensors	Use of indium phosphide (InP) to detect biomaterials Functionalization and covalent biomolecule immobilization	High sensitivity and reproducibility for DNA and protein detection	*Xylella fastidiosa*, Citrus Tristeza Virus (CTV)	Moreau et al. (2012)
Carbon nanotube-based sensors	Vacuum filtration to deposit CNTs on paper with metal masks Control over dimensions through mask design	Low-cost, sensitive, and suitable for pH and chemical detection	General chemical and biomolecular analysis	Lei et al. (2012)
Silicon nanowire sensors	CMOS-compatible top-down fabrication using electron beam lithography Chemical modification for DNA hybridization detection	High sensitivity for biomolecule detection with precise surface modification	DNA-based phytopathogen detection	Abd Rahman et al. (2022)

**Figure 5:**
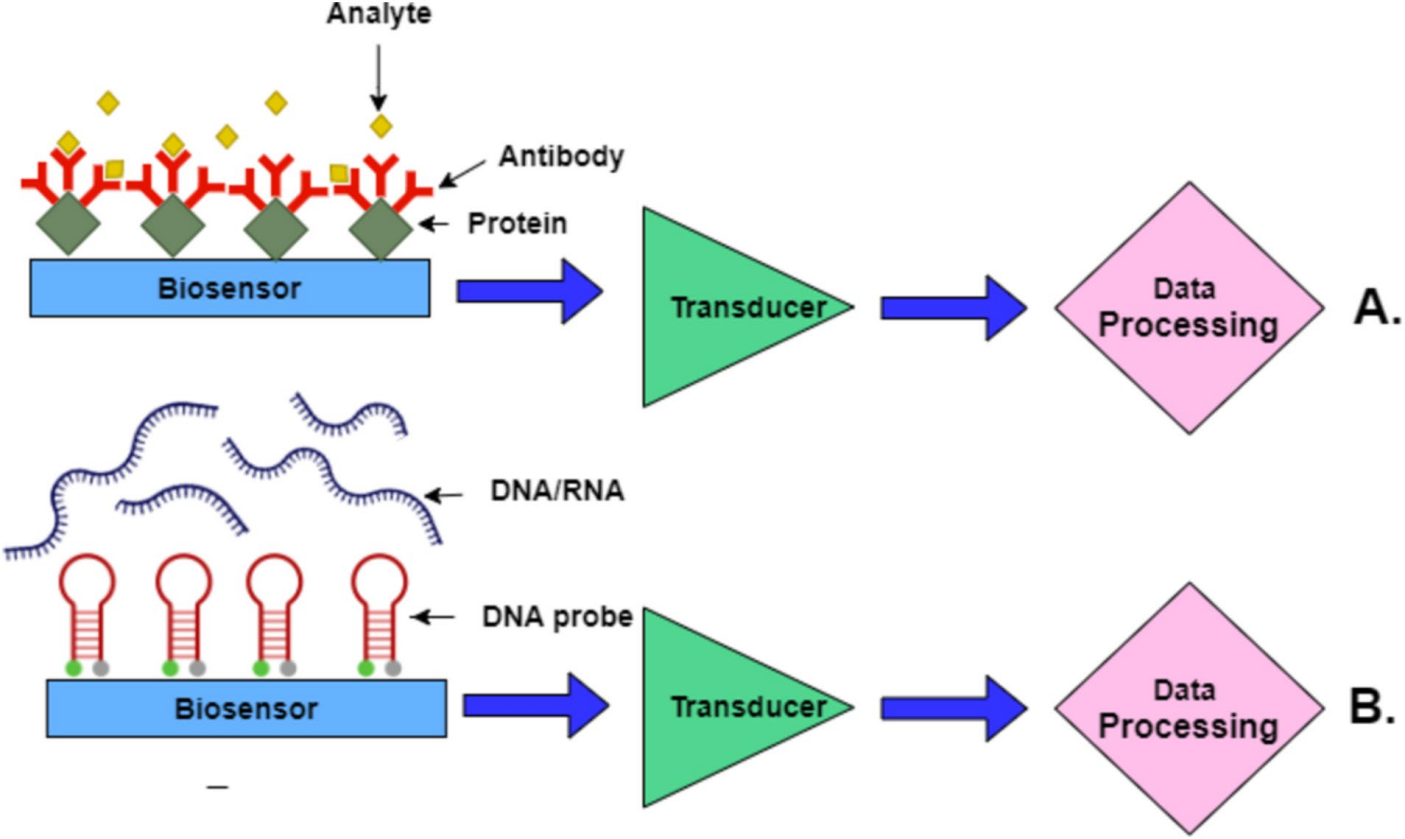
Schematic representation of biosensor mechanisms. **(A)** Immunosensor-based mechanism. **(B)** Nucleic acid-based biosensor mechanism.

Figure 5 presents two key biosensor mechanisms. In part A, the top schematic illustrates the interaction in an immunosensor where antibodies bind specifically to target proteins (analyte). This interaction is detected by a transducer, which processes the signal for data analysis. In part B, the bottom schematic shows a nucleic acid biosensor where DNA/RNA sequences interact with complementary DNA probes on the biosensor surface. The interaction generates a signal processed through the transducer, similar to immunosensors, to detect the presence of specific nucleic acids.

**Figure 6:**
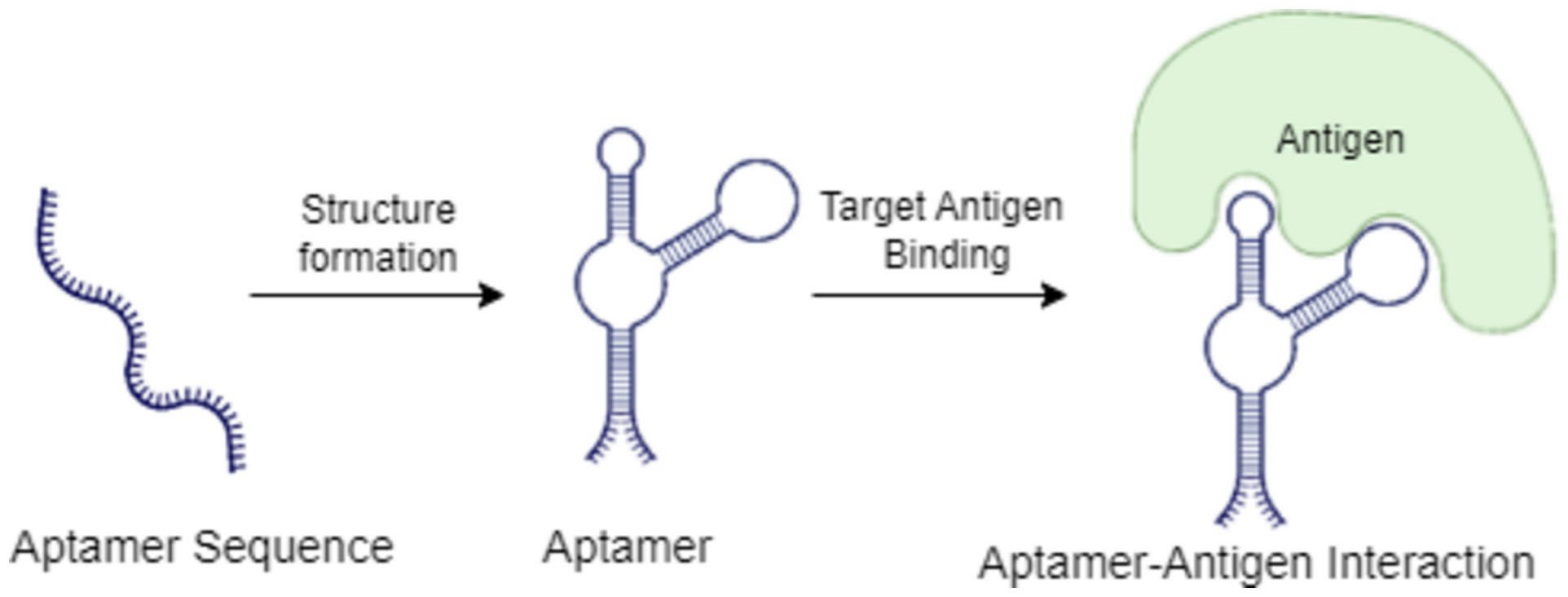
Mechanism of aptamer-antigen interaction in biosensor applications.

Figure 6 illustrates the mechanism of aptamer-antigen interaction in biosensor applications. The process begins with an aptamer sequence, which forms a specific structure through folding. Once the aptamer assumes its active conformation, it selectively binds to the target antigen, creating a stable aptamer-antigen complex. This interaction is crucial in biosensor applications, as it triggers a detectable signal that can be used to identify and quantify specific pathogens or analytes in various agricultural and environmental samples.

**Figure 7:**
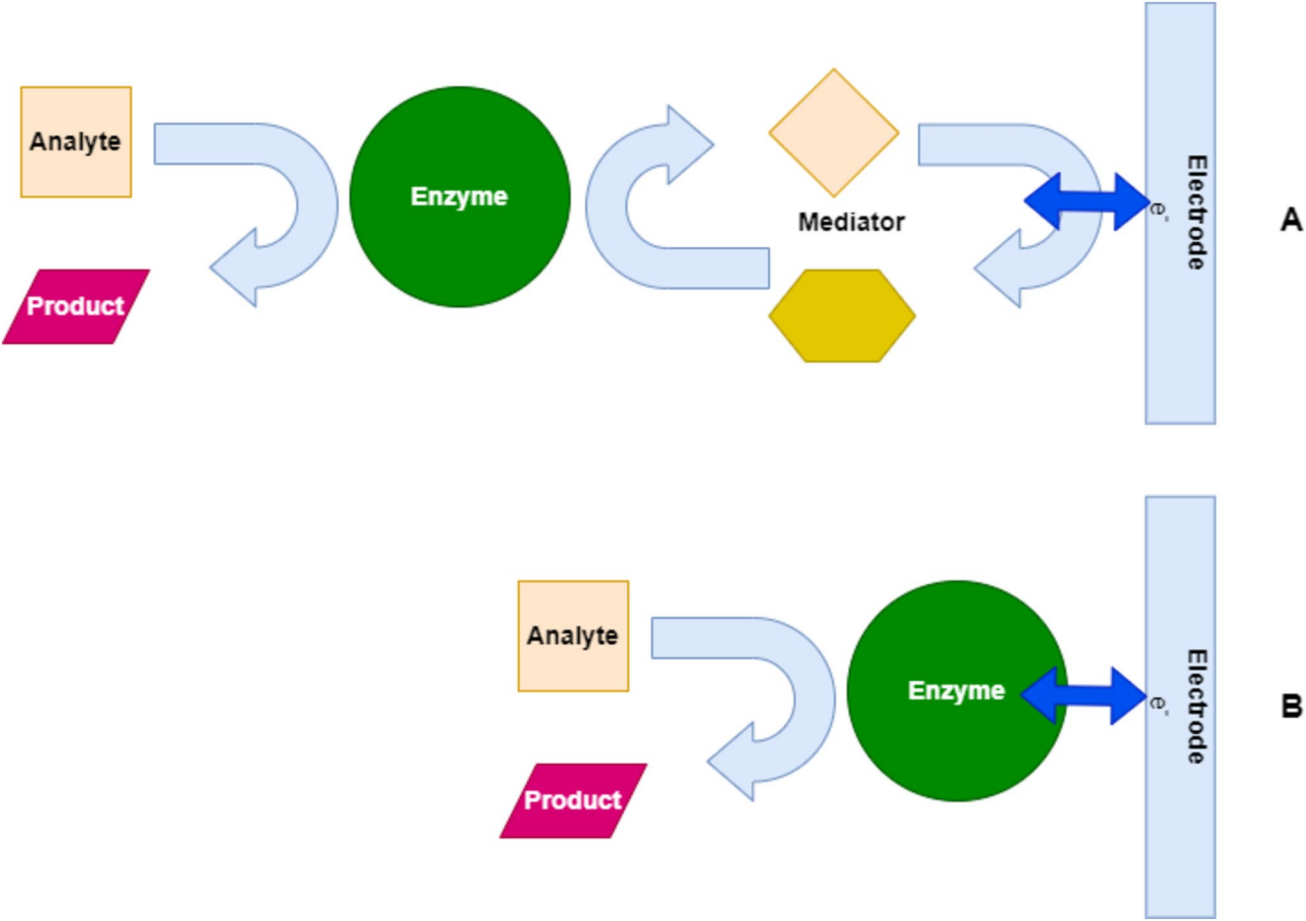
Mechanism of enzyme-based biosensors for pathogen detection. **(A)** Mediator-assisted enzyme-based biosensor. **(B)** Direct enzyme-based biosensor.

Figure 7 illustrates the operational mechanism of enzyme-based electrochemical biosensors for pathogen detection. In Pathway A, the enzyme catalyzes the conversion of an analyte into a product with the help of a mediator, which facilitates electron transfer to the electrode, generating a detectable signal. In Pathway B, the direct interaction between the enzyme and the electrode without a mediator results in the same outcome, demonstrating two common approaches used in biosensor technology.

##### 2.3.1.1 Optical biosensors

Optical biosensors detect changes in optical properties such as absorbance, fluorescence, or refractive index upon interaction with the target pathogen. Surface plasmon resonance (SPR) biosensors have been employed for label-free detection of plant viruses like Tobacco Mosaic Virus (TMV), offering real-time monitoring and high sensitivity (Hariharan and Prasannath, 2021). Surface SPR biosensors are increasingly used for the label-free and highly sensitive detection of various plant viruses beyond TMV. These sensors provide real-time monitoring and can be specifically tailored to detect various plant viral pathogens. An SPR biosensor has been developed to detect MCMV by utilizing a gold surface coated with 11-mercaptoundecanoic acid, which is then functionalized with anti-MCMV antibodies. This setup achieved a detection limit of around 1 part per billion (ppb), showcasing its high sensitivity compared to conventional methods like ELISA (Zeng et al., 2013). In another application, SPR biosensors were used to monitor the interaction of Potato Virus Y with monoclonal antibodies, allowing researchers to study various serotypes and their interactions with antibodies in real-time. This biosensor setup was beneficial for optimizing serologic diagnostic tools and enhancing our understanding of viral serotype variability (Gutiérrez-Aguirre et al., 2014).

**Figure 8:**
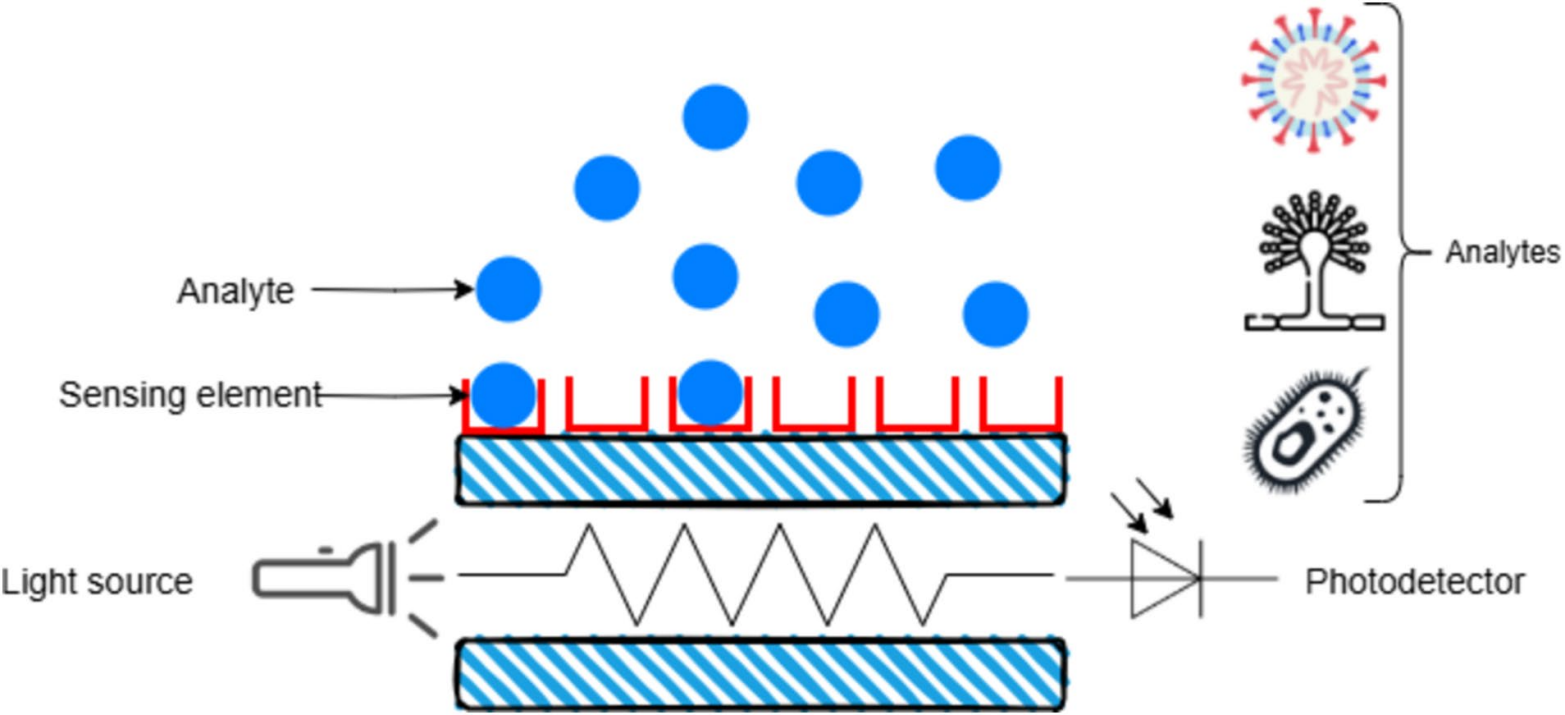
Working principle of optical biosensor.

Fluorescence-based sensors utilize fluorescent markers that emit signals upon binding to the target pathogen. Portable fluorescence detectors have been developed to detect pathogens like *Erwinia amylovora*, the causative agent of fire blight in apples and pears. Figure 8 depicts an optical sensor where an analyte interacts with a sensing element, altering light from a source, which is then detected by a photodetector to measure the analyte.

##### 2.3.1.2 Electrochemical biosensors

Electrochemical biosensors, which detect changes in electrical signals caused by biochemical reactions, have emerged as powerful tools for detecting pathogens in agriculture. By incorporating nanomaterials like gold nanoparticles, carbon nanotubes, and quantum dots, these sensors have significantly advanced in-field diagnostics, offering rapid and sensitive detection of phytopathogens.

Gold nanoparticles, widely known for their signal-amplifying properties, play a key role in lateral flow assays. These nanoparticles bind to pathogen-specific antibodies, enabling quick, on-site diagnostics without lab-based testing. For instance, they have been effectively used to detect *Phytophthora infestans*, a major pathogen responsible for potato blight, supporting disease management directly in the field (Bobrinetskiy et al., 2021; Kashyap et al., 2016b). Similarly, carbon nanotubes, prized for their high conductivity, enhance signal transduction in biosensors, making them particularly useful in detecting viral pathogens like the Tomato Yellow Leaf Curl Virus. These nanotubes detect even minute concentrations of viral DNA, crucial for early intervention and preventing widespread outbreaks (Rajwade et al., 2020; Attaluri and Dharavath, 2023).

Another innovative approach involves the use of quantum dots in smartphone-integrated biosensors. These quantum dots fluoresce when they interact with target genetic material, and the fluorescence is captured and analyzed by smartphone cameras, offering instant and quantitative pathogen assessments. This technology, used to detect fungal pathogens like *Fusarium oxysporum*, enables rapid decision-making and facilitates data sharing across networks, improving coordinated responses to agricultural threats (Rajwade et al., 2020; Attaluri and Dharavath, 2023).

Enzyme-linked biosensors are another vital tool for field diagnostics. These sensors utilize enzyme-catalyzed reactions to produce measurable signals, such as colorimetric or electrochemical outputs, allowing on-site detection without specialized lab equipment. Miniaturized versions of ELISA are widely used to detect viruses such as Plum Pox Virus in stone fruits and Maize Chlorotic Mottle Virus in maize. By linking pathogen-specific antibodies to enzymes that produce a visible color change upon pathogen detection, these devices offer real-time monitoring capabilities with sensitivity comparable to PCR, making them essential for prompt agricultural interventions (Ali et al., 2021; Ray et al., 1856).

Beyond viral detection, enzyme-linked biosensors have also proven effective in identifying bacterial and fungal pathogens, such as *Fusarium oxysporum*, which causes wilt disease in tomatoes. These sensors utilize peroxidase-based reactions to generate optical signals upon detecting fungal markers, making them ideal for on-site testing in remote agricultural areas where lab access is limited. Additionally, these biosensors are also capable of monitoring organophosphate pesticide residues. Acetylcholinesterase-based sensors detect pesticide residues by measuring enzyme inhibition, providing a dual benefit of pathogen and pest monitoring, which is crucial for sustainable agricultural practices (Ali et al., 2021; Kaur and Singh, 2020; Karadurmus et al., 2021).

**Figure 9:**
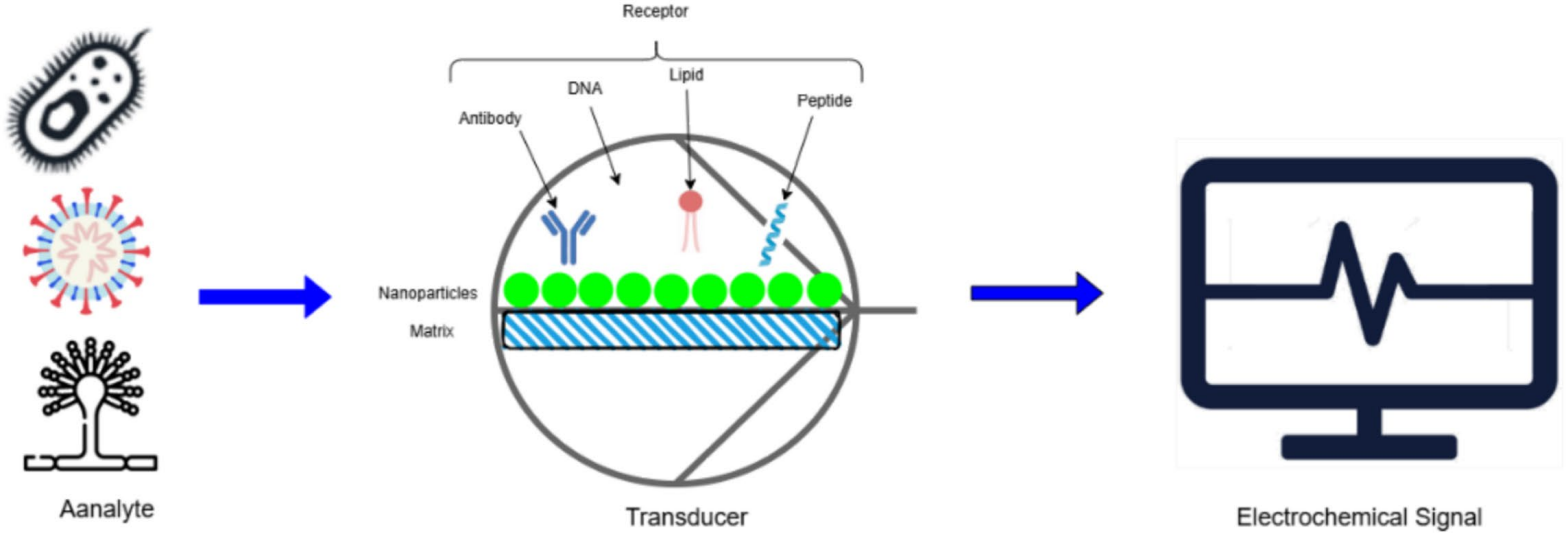
Schematic representation of electrochemical biosensor for phytopathogen detection.

Figure 9 illustrates an electrochemical biosensor designed for phytopathogen detection. The analyte, such as microbial pathogens or their markers, interacts with a specific receptor (antibody, DNA, lipid, or peptide) immobilized on a nanoparticle matrix. This interaction generates a signal transduced into an electrochemical output, enabling pathogen identification and quantification.

#### 2.3.2 Nucleic acid amplification techniques

##### 2.3.2.1 Isothermal amplification methods

**Figure 10:**
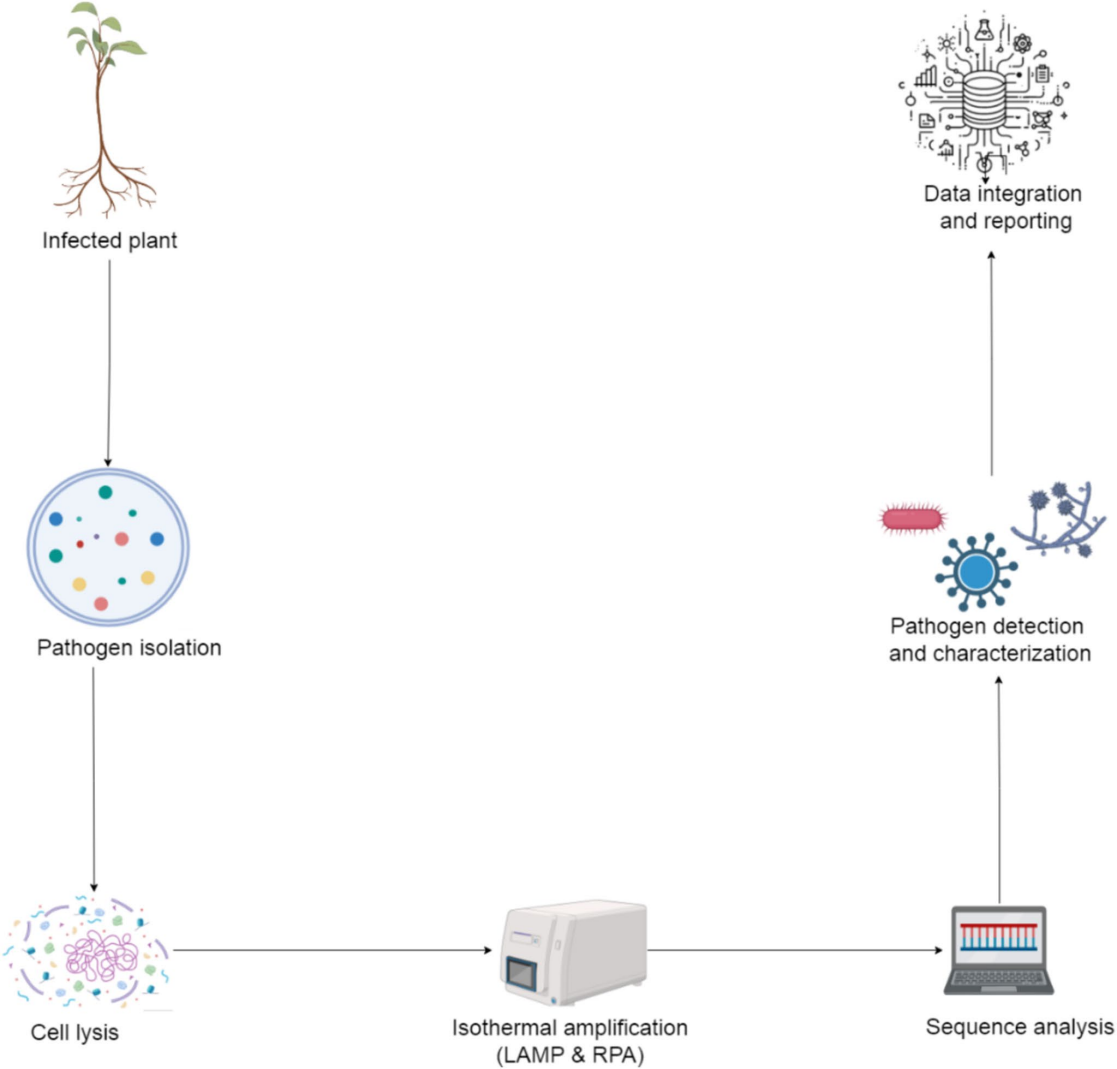
Workflow of portable isothermal nucleic acid amplification method.

Isothermal nucleic acid amplification techniques, like LAMP and recombinase polymerase amplification (RPA), offer the advantage of operating at constant temperatures, eliminating the need for complex thermal cycling equipment, characteristic of PCR-based methods. These techniques are increasingly integrated into portable, field-deployable devices for rapid diagnostics. For instance, LAMP assays have been successfully used in portable formats to detect plant pathogens like *Phytophthora infestans*, the causative agent of late blight in potatoes, with amplification times usually under 30 min and colorimetric readouts for ease of interpretation (Ristaino et al., 2020). Similarly, RPA technology, operating at low temperatures (37–42°C), has been employed in handheld devices for the detection of viruses, such as the Tomato Yellow Leaf Curl Virus (TYLCV), making it highly suitable for field diagnostics (Wang and Yang, 2019). Figure 10 illustrates a detailed workflow for portable isothermal nucleic acid amplification techniques, including sample collection, preparation (involving cell lysis and nucleic acid purification), and the essential primer binding step for amplification.

##### 2.3.2.2 PCR-based portable devices

PCR is a critical molecular biology technique known for its high specificity and sensitivity in nucleic acid amplification and pathogen detection. Traditionally confined to centralized labs due to the complexity of thermal cycling and real-time detection, recent advancements in microfluidics, miniaturization, and power efficiency have created portable PCR devices. Portable PCR devices replicate the core functions of conventional machines. Still, they are optimized for field use through features such as battery-operated thermal cyclers and microfluidic chambers, which enable rapid temperature cycling and efficient reagent use. Integrated fluorescence detection further allows real-time monitoring of DNA amplification, making quantitative PCR (qPCR) feasible in non-laboratory environments. These innovations have made portable PCR invaluable for detecting pathogens, including plant and bacterial contaminants, especially in on-site diagnostics and environmental assessments (Koo et al., 2013).

One notable example of these advancements is the handheld qPCR device, which offers high specificity and sensitivity for detecting plant pathogens on-site. This compact and user-friendly tool is particularly beneficial in agriculture, where early detection of diseases can prevent significant crop losses (DeShields et al., 2018). Additionally, devices such as the BioFire FilmArray system integrate multiple processes, from nucleic acid extraction to real-time PCR, into a single platform, delivering results in under an hour, demonstrating the potential of portable PCR devices in decentralized pathogen detection (Pham et al., 2024).

**Figure 11:**
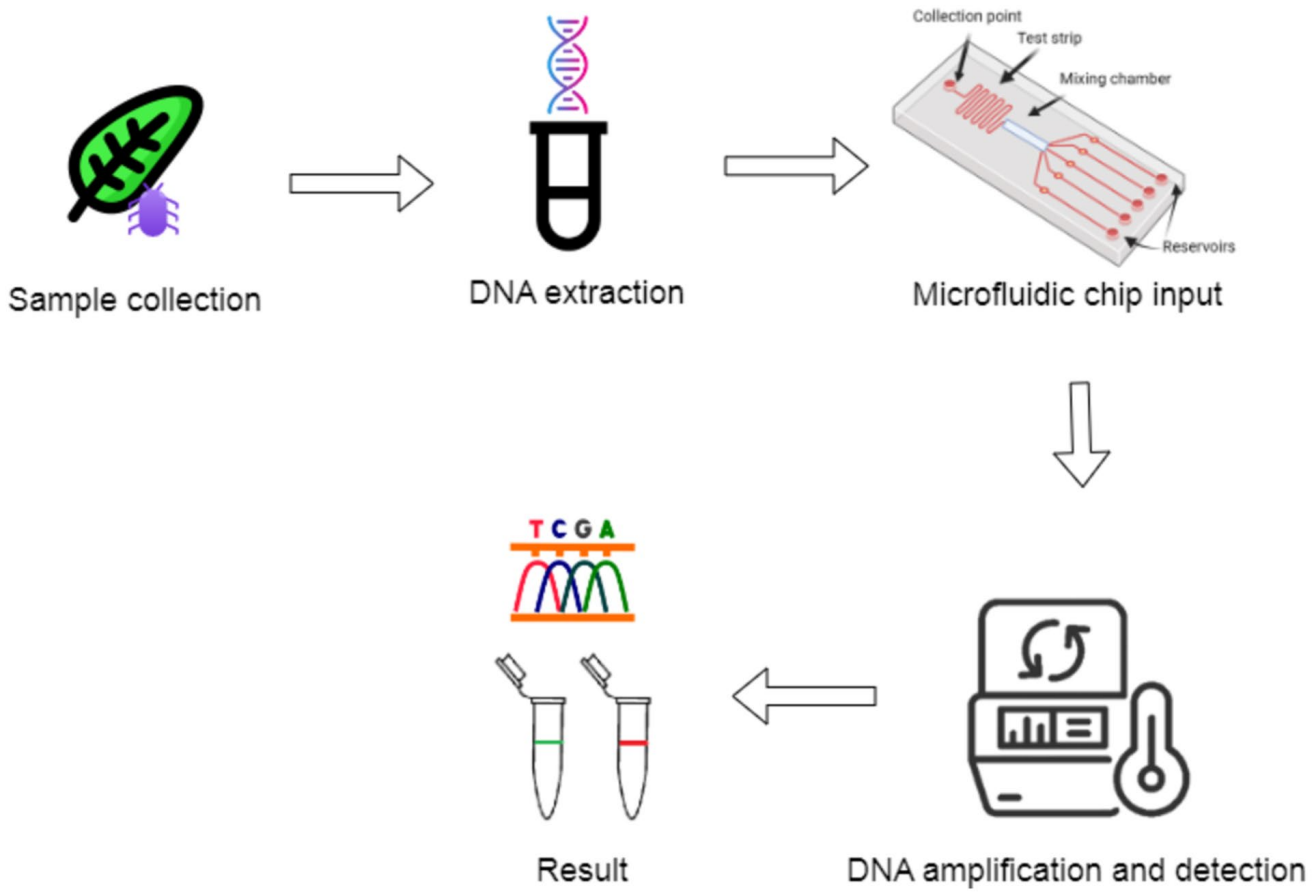
Portable PCR workflow with the integration of microfluidics for rapid DNA amplification and detection.

Field-based PCR and other molecular devices have revolutionized pathogen detection by providing real-time diagnostics in agricultural settings (Figure 11). These tools allow rapid identification of diseases, enabling timely intervention. Devices like the miniPCR, a compact thermocycler, have been effectively used to detect *Phytophthora infestans*, the causative agent of potato late blight, providing lab-equivalent results in minutes. The miniPCR connects to smartphones or tablets for real-time result monitoring, crucial for field-based diagnostics in remote areas (Gonzalez-Gonzalez et al., 2020; Ssengo et al., 2020). Similarly, the Biomeme two3 platform integrates qPCR with smartphone technology, simultaneously supporting up to three reactions and allowing real-time pathogen detection, such as *Fusarium oxysporum*, within 45 min. The system’s smartphone interface facilitates remote data sharing and decision-making, enhancing its utility in the field (Buja et al., 2021; Marx, 2015).

#### 2.3.3 Immunoassay-based portable analyzers

Immunoassays leverage the specificity of antigen-antibody interactions for pathogen detection. Lateral Flow Immunoassays (LFIA) are commonly used for their simplicity and rapid results. Portable devices utilizing LFIA have been developed for on-site detection of various plant pathogens, including *Xanthomonas* species in citrus plants (Srinivasan and Tung, 2015). These assays provide results within minutes and are user-friendly, requiring minimal training.

**Figure 12:**
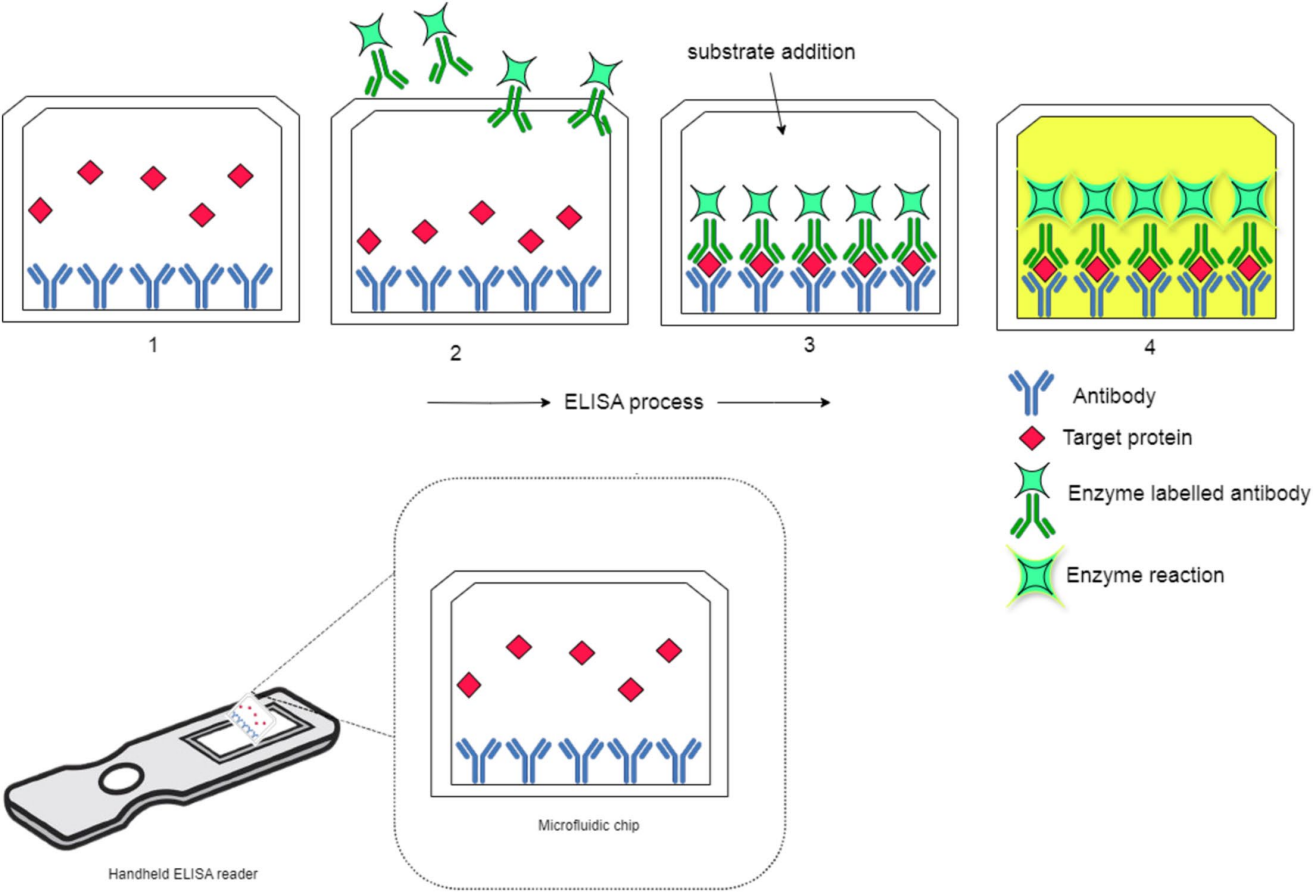
Schematic representation of the portable ELISA process.

Figure 12 illustrates the portable ELISA process, operating on the same principle as LFIA, utilizing antigen-antibody interactions for pathogen detection. In ELISA, antibodies are first immobilized on the plate to capture target proteins, similar to how LFIA detects pathogens like *Xanthomonas* species in citrus plants. This cost-effective and widely used technique ensures selective binding, essential for accurate detection. The target protein binds to the immobilized antibodies, reflecting the precise interaction seen in LFIA. A secondary enzyme-conjugated antibody then binds to a different epitope, forming a sandwich structure that mirrors the detection phase of LFIA. Finally, a substrate is added, reacting with the enzyme to produce a visible color change, confirming the presence of the target. This rapid detection is akin to LFIA’s quick results in field applications. The figure effectively shows how ELISA and LFIA, though different in their settings, share the same core immunoassay principles for pathogen detection.

**Figure 13:**
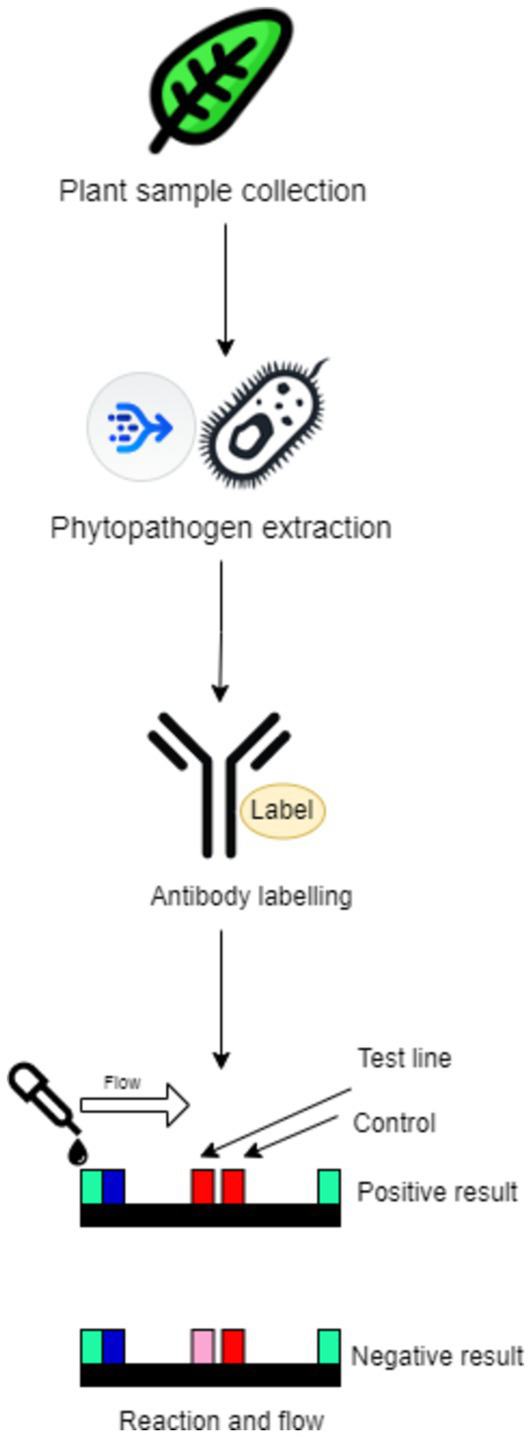
Example of a portable lateral flow immunoassay for pathogen detection, showing the test and control lines in positive and negative results.

Portal lateral flow assays (LFAs) and immunoassay devices are vital for rapid and cost-effective phytopathogen detection. With the increasing importance of timely diagnostics in agriculture, these devices have shifted diagnostics from the laboratory to the field, allowing farmers and agronomists to implement real-time solutions. Their simplicity, portability, and quick result delivery make LFIAs ideal for field-based diagnostics, while recent advancements in sensitivity have expanded their utility in agriculture (Figure 13). Using nanoparticles like gold and quantum dots enhances the assay signal, allowing the detection of even minimal pathogen concentrations, such as in the detection of potato virus X through nanoparticle enlargement, which lowered detection limits and enhanced precision (Banerjee and Jaiswal, 2018). Another innovation involves graphene oxide, which improves sensitivity through photoluminescence, enabling the detection of pathogens like *Escherichia coli*, suggesting potential for a range of phytopathogen applications (Avila-Huerta et al., 2020).

A significant advancement is the incorporation of CRISPR technology into lateral flow diagnostics. The CRISPR-based Bio-SCAN LFA allows for rapid detection of plant pathogens and transgenic elements within an hour. This field-deployable system has been tested on crops like wheat and rice, proving effective for disease management and crop breeding (Ali et al., 2022). Additionally, nucleic acid-based LFAs with RPA technology present a promising approach for viral pathogen detection. This method has demonstrated PCR-comparable sensitivity in detecting potato virus X and is suitable for rapid, on-site applications that require swift results (Ivanov et al., 2020).

Innovations aimed at enhancing assay sensitivity continue to drive progress. For instance, post-assay nanoparticle growth techniques have achieved much lower detection limits, allowing precise diagnostics for even small quantities of target analytes. Techniques such as multicolor signal integration on a single assay line enable the simultaneous detection of multiple pathogens, increasing test efficiency (Di Nardo et al., 2019). In addition, time-resolved luminescent nanoparticles extend these devices’ dynamic range and sensitivity, making them suitable for more complex field conditions (Song and Knotts, 2008).

In the context of phytopathogen diagnostics, aptasensors can be tailored to identify specific microbial or fungal markers associated with plant pathogens. By leveraging MXene’s excellent electrical conductivity, high surface area, and tunable properties, these sensors can achieve high sensitivity and specificity. Integrating aptamers into MXene-based platforms further enables rapid, cost-effective, and portable detection methods that are particularly advantageous for on-site plant disease diagnostics (Parihar et al., 2022).

MXene-based aptasensors also show significant promise for detecting phytopathogenic toxins, such as aflatoxins, in agricultural and food matrices. Their high electrical conductivity, biocompatibility, and customizable surface features facilitate quick, sensitive, and affordable toxin detection. This approach surmounts the limitations of conventional methods—like HPLC or ELISA—by offering faster and simpler analytical workflows. When combined with IoT and advanced data analytics, these portable systems enable real-time monitoring to ensure crop health and food safety (Parihar et al., 2023).

Building upon these advancements, the novel approach of electrochemical aptasensor fabrication for phytopathogen biomarker detection focuses on synthesizing a reduced graphene oxide-yttrium nanocomposite and employing it in electrochemical aptasensor construction. By integrating phytopathogen-specific aptamers, this technique delivers a label-free, highly sensitive, and cost-effective diagnostic platform. Critically, this strategy supports the early detection of plant diseases, making it an instrumental tool in strengthening agricultural diagnostics and improving overall crop health management (Parihar and Khan, 2024).

## 3 Data integration and digital connectivity

Advancements in digital technologies, particularly the Internet of Things (IoT) and artificial intelligence (AI), have revolutionized phytopathogen detection, offering real-time, field-based solutions that enhance precision agriculture. Integrating IoT with portable diagnostic methods enables seamless data collection and transmission from diagnostic devices to centralized systems, ensuring faster decision-making and improved disease management strategies.

### 3.1 Role of IoT and cloud-based analytics

**Figure 14:**
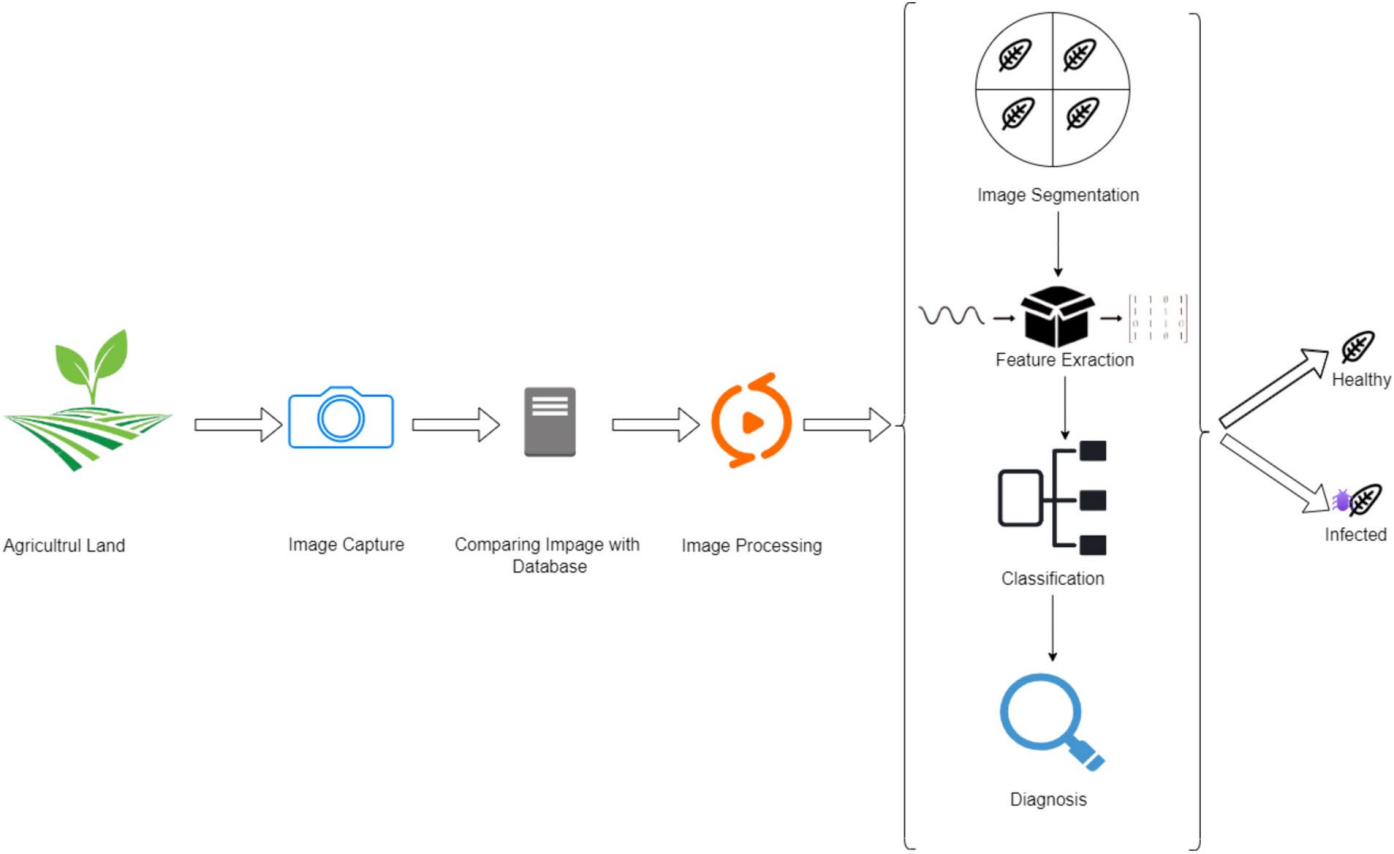
Internet of Things (IoT) in plant pathogen detection.

The integration of IoT in agriculture has fundamentally improved the ability to monitor and manage agricultural practices with real-time, actionable data. This technology enables portable devices like PCR, LAMP, and ELISA to detect phytopathogens directly in the field, transmitting data to cloud systems for analysis and alerts. By facilitating immediate intervention through mobile alerts, IoT-connected devices help farmers take proactive steps to contain diseases such as *Fusarium* wilt, as demonstrated in successful applications across global agricultural settings (Dholu and Ghodinde, 2018; Kumar et al., 2023). Figure 14 describes the application of IoT in plant pathogen detection.

IoT-enabled devices have optimized pathogen detection in vineyards, reducing pesticide use while maintaining crop quality (Trilles et al., 2020). In Southeast Asia, IoT-based LAMP systems detected bacterial blight in rice, providing timely data in regions with limited lab access and enhancing regional pathogen tracking (Uddin et al., 2017). Similarly, U.S. farmers managing soybean rust have benefited from IoT kits that enabled targeted fungicide applications (Ayaz et al., 2019).

Beyond pathogen detection, IoT’s role in agriculture includes granular data collection supporting predictive models for disease prevention. However, challenges such as costs, connectivity issues in rural areas, and technical expertise remain significant obstacles to its broader adoption (Khuwaja et al., 2023; Siskandar et al., 2020). Nevertheless, IoT is pivotal in shaping resilient and data-driven farming practices, optimizing resource use, and ensuring sustainable agricultural operations.

### 3.2 Artificial intelligence and machine learning for data interpretation

Applying AI and Machine Learning (ML) in agriculture, particularly in portable diagnostic tools, has transformed how diseases are managed in real-time. AI-driven platforms, including PlantVillage Nuru and, ViT-SmartAgri harness the potential of frameworks like TensorFlow and PyTorch to provide farmers with rapid and precise disease diagnostics via smartphones. These tools empower smallholder farmers worldwide, reducing dependency on costly lab tests and enhancing disease management.

For example, PlantVillage Nuru identifies plant diseases with over 90% accuracy by analyzing smartphone images of leaves, enabling farmers in East Africa to mitigate crop losses effectively (Mrisho et al., 2020). ViT-SmartAgri leverages advanced vision transformer models to detect subtle symptoms in crops like tomatoes, further supporting precision agriculture with highly accurate disease identification (Boukabouya et al., 2022).

In addition to image-based diagnostics, AI algorithms such as Support Vector Machines (SVMs) and k-NN classifiers are being used in multiplex detection systems to classify pathogens like *Ralstonia solanacearum*, improving the speed and accuracy of field-based diagnostics (Ryo et al., 2009). Predictive modeling through platforms integrating IoT and AI further supports preemptive disease management. These models, such as Random Forest and RNNs, predict outbreaks of diseases like potato late blight, enabling farmers to implement preventive strategies before infections spread (Meno et al., 2023).

With millions of global users, apps like Plantix, which diagnose plant diseases and nutrient deficiencies, have significantly contributed to sustainable farming practices (Zhao et al., 2024).

Moreover, portable spectrometers combined with AI algorithms, such as Decision Trees and Support Vector Machines, analyze plant nutrient deficiencies by interpreting leaf reflectance spectra, providing farmers with immediate insights to optimize plant health (Li D. et al., 2020). AI models, including LSTM networks, are also used for forecasting pest outbreaks based on climatic data, allowing for targeted interventions (Chen et al., 2022). Drone-based systems equipped with AI offer large-scale crop monitoring, enabling efficient management of disease hotspots in expansive agricultural fields (Bawa et al., 2024).

### 3.3 Data management, connectivity and smartphone applications

These portable diagnostic devices generate diverse, real-time data streams—from diagnostic results to geospatial coordinates and temporal disease trends—and rely on robust pipelines for harmonization, analysis, and dissemination. Wireless connectivity through Wi-Fi, LPWANs, and 4G/5G networks ensures seamless data transfer to cloud servers and decision-support tools, enabling systems to integrate with nanopore sequencing for real-time pathogen analysis, machine learning-driven outbreak predictions and targeted fungicide recommendations (Fountas et al., 2020). Interoperable data formats and standardized protocols promoted by organizations like ISO facilitate large-scale disease surveillance, informed by spatial data on virulence races of pathogens such as wheat rust (Gilligan, 2024). Embedded algorithms enhance data quality control, while smartphone-integrated microfluidic chips ensure accurate detection by adjusting for environmental variables (Chen et al., 2017). Connectivity also supports collaborative diagnostics, allowing remote consultation with experts and global data-sharing for emerging pathogens (Lau and Botella, 2016), and CRISPR-based devices coupled with geospatial data can predict disease spread to optimize management strategies (Morcia et al., 2023). Data security measures, including encryption and authentication, safeguard agronomic information, while AI models and multi-modal analyses combining external data sources, like satellite imagery and soil profiles, support holistic disease management (Mahlein, 2016).

**Figure 15:**
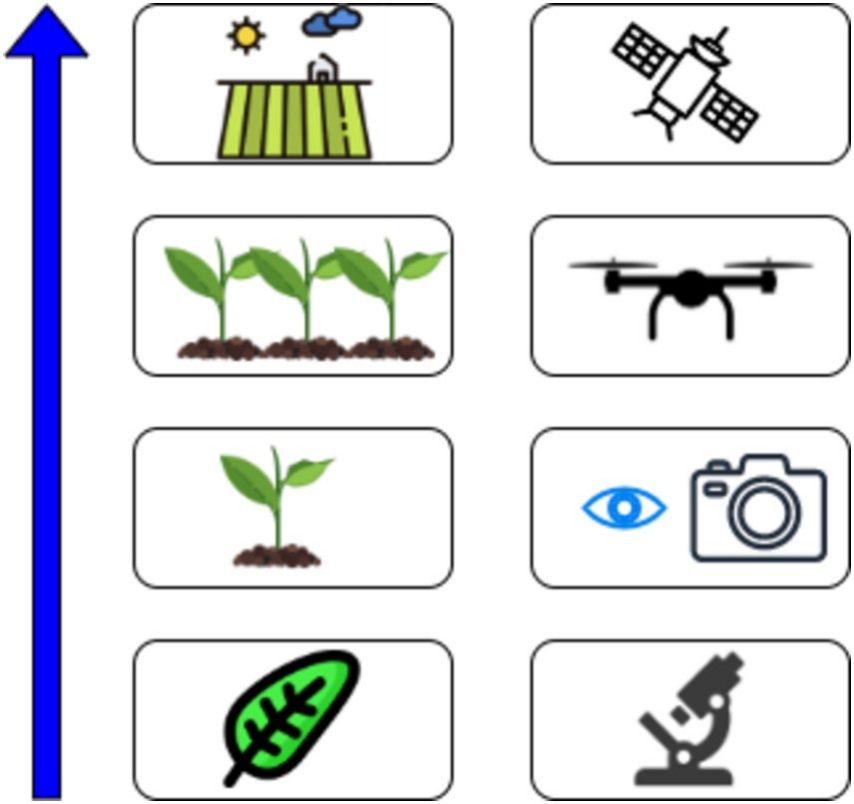
Schematic representation of multiscale non-invasive spectral and optical techniques for detecting biotic stresses in plant parts, individual plants, and field-level systems.

Flexible connectivity frameworks integrate evolving diagnostic technologies—CRISPR-based tools, synthetic biology biosensors—into existing IoT and cloud platforms, benefiting large-scale and smallholder agriculture. For instance, China’s IoT-based system for crop disease prevention uses ozone sterilization and light-trap technology, transmitting environmental and pathogen data to a central system for real-time alerts and reducing chemical inputs (Wang et al., 2024). Cloud-based analytics platforms like Arable and Semios combine sensor data on plant health, pests, and weather conditions, enabling immediate, data-driven insights and collaboration among farmers, extension services, and researchers. Large-scale epidemiological analysis and predictive modeling help manage infections like *Xylella fastidiosa* in olive orchards (Hornero et al., 2020) and predict *Phytophthora infestans* outbreaks in potatoes using machine learning models (Duarte-Carvajalino et al., 2018). This convergence of wireless sensor networks, cloud analytics, and AI-driven modeling fundamentally enhances real-time decision-making, resilience, and sustainable disease control, empowering modern agriculture to confront climate change and evolving pathogen challenges. Figure 15 illustrates the resolution of various plant stress detection techniques.

### 3.4 Smartphone applications

Smartphone applications for phytopathogen detection have revolutionized agricultural diagnostics, leveraging AI and machine learning to provide real-time disease management solutions. Apps like Plantix are widely used in South Asia and Africa, enabling farmers to diagnose diseases like powdery mildew and rust through image analysis. Plantix’s extensive database covers over 30 crops and 400 diseases, and it also offers a community feature for farmers to share insights, making it a valuable resource in regions with limited access to traditional agricultural support (Samal et al., 2023).

AgroAI targets staple crops such as rice and maize, using CNNs to identify bacterial blight and maize rust while also forecasting disease outbreaks based on environmental data like humidity and temperature (Terentev and Dolzhenko, 2023). AgroAI’s predictive capabilities allow farmers to implement preventive measures, reducing crop losses and improving crop health. In East Africa, iCassava addresses cassava diseases like the Cassava Brown Streak Virus (Enkvetchakul and Surinta, 2022). Developed with TensorFlow’s mobile framework, iCassava, a computer vision dataset, operates offline, essential in remote areas of Uganda and Tanzania. Field tests have demonstrated high accuracy, bolstering efforts to protect this staple crop from devastating diseases (Mwebaze et al., 2019).

DetectPlant uses the EfficientNetV2B2 model to detect diseases in tomatoes and grapes, achieving high accuracy through transfer learning, which adapts pre-trained models to specific crops (Debnath et al., 2023). Meanwhile, CropDoctor is widely adopted in India to identify diseases and nutrient deficiencies across crops like wheat and soybeans. Its AI-powered analysis provides farmers with actionable recommendations, and studies have shown that its guidance can increase crop yields by up to 15% (Siddiqua et al., 1869). LeafDoctor serves researchers and extension officers by quantifying disease severity in crops like wheat and corn. By estimating the percentage of leaf area affected, LeafDoctor helps monitor the effectiveness of treatment strategies, facilitating more precise disease management decisions over time (Maginga et al., 2024). Table 3 summarizes various smartphone applications used in phytopathogen detection.

**Table 3:** Summary of smartphone applications for plant disease detection and management.

App name	Description	References
Agrio	Offers plant disease diagnosis through image analysis, provides alerts and connects users with agronomists for advice	Siddiqua et al. (1869)
AgroScout	Uses drone and AI technology to monitor fields and detect early signs of disease	The AgroScout (2024)
Crop Doctor	A diagnostic tool for identifying diseases in various crops and suggesting management practices	Mandal et al. (2022)
CropsAI	Detects diseases in various crops, offers predictions for disease spread based on climatic conditions, and suggests remedies	Yao (2024)
FarmRise	Provides crop advisory services, including pest and disease detection. Covers a wide range of crops. Available in multiple languages	Praneeth (2023)
iScoute	An app that leverages AI for real-time disease and pest detection in crops	Calle and Böckmann (2020)
Leaf Doctor	App that quantifies the severity of plant disease symptoms by analyzing leaf images	de Almeida et al. (2021)
MajraDoc	An image-based disease detection app for agricultural plants using deep learning techniques	Alfozan and Hassan (2021)
mPD	Diseases diagnosis application using convolutional neural network	Asani et al. (2023)
MyPestGuide	Developed by the Department of Primary Industries and Regional Development in Western Australia, it helps identify and report pests and diseases	Wright et al. (2018)
FieldClimate	Provides disease models and forecasts to help farmers anticipate and manage plant diseases	METOS (2005), Enkvetchakul and Surinta (2022), Mwebaze et al. (2019), Enkvetchakul and Surinta (2022), Terentev and Dolzhenko (2023), and Gilligan (2024)
Pestoz	Assists in the identification of pests and diseases affecting various crops	Siddiqua et al. (1869)
Plante	An app that helps in diagnosing plant health issues through image analysis	Oliveira et al. (2020)
Plantix	A crop doctor app that helps identify diseases, pests, and nutrient deficiencies across various crops	Samal et al. (2023)
PlantSnap	Identifies plant species and provides disease diagnostics for over 600,000 species. Community of users for data and feedback exchange	Bawingan et al. (2024)
Rice Doctor	Created by the International Rice Research Institute, this app helps farmers diagnose diseases, pests, and nutrient deficiencies in rice	Pascual et al. (2020)

### 3.5 Application in disease surveillance and management

Portable diagnostic devices have revolutionized disease surveillance by enabling real-time monitoring directly within agricultural settings. These devices integrated into routine farming practices allow for continuous crop monitoring, crucial for early detection and rapid intervention against fast-spreading pathogens.

The data collected by these portable diagnostics is key to data-driven decision-making, enabling precise, targeted disease management. Field-based diagnostic data help farmers and agricultural managers make informed decisions about interventions, such as targeted fungicide application, removal of infected plants, or effective quarantine measures. For example, information on *Xylella fastidiosa* infections in olive groves informs selective pruning and vector control strategies (Amoia et al., 2023). Additionally, geotagged diagnostic data can be integrated into regional systems, allowing agricultural networks to track and respond to disease trends more effectively. By mapping infection patterns, stakeholders can anticipate future outbreaks and implement preemptive measures.

## 4 Applications across diverse plant pathogens

The advancements in handheld technologies—from LAMP-based assays and lateral flow immunoassays (LFIAs) to microfluidic LOC platforms and smartphone-integrated systems—have transformed plant disease diagnostics. In contrast to traditional laboratory-based methods, these compact devices offer rapid, accurate, and cost-effective on-site identification of pathogens, enabling farmers and agronomists to implement immediate control measures. The text underscores how these novel approaches help mitigate substantial crop losses, strengthen food security, and streamline disease management practices. The discussion underlines the importance of portable diagnostics as a cornerstone of sustainable plant health strategies by connecting recent technological breakthroughs to their practical field applications.

### 4.1 Diagnostic approaches for major pathogen groups

Accurate and timely diagnosis of plant pathogens is critical to minimizing agricultural losses and safeguarding global food security. While reliable, traditional diagnostic methods often require extensive laboratory infrastructure, technical expertise, and significant time, limiting their utility in the field. Advances in portable, rapid, and user-friendly diagnostic tools are revolutionizing the management of major pathogen groups, enabling farmers and agronomists to take swift action at the point of need. These novel approaches leverage cutting-edge technologies such as microfluidic LOC platforms, LFIAs, and smartphone-integrated detection systems to provide accessible and precise solutions for pathogen detection.

Table 4 provides a comprehensive list of significant plant pathogens, categorized by their type and primary target host plants. This summary highlights the diverse pathogens affecting key crops and underscores the need for targeted diagnostic and management strategies.

**Table 4:** List of some significant plant pathogens with their target host.

Pathogen name	Category	Target plants	References
*Botrytis cinerea*	Fungi	Grapes, strawberries, tomatoes	Dean et al. (2012)
*Fusarium graminearum*	Fungi	Cereals	Dean et al. (2012)
*Fusarium oxysporum*	Fungi	Tomatoes, bananas	Dean et al. (2012)
*Magnaporthe oryzae*	Fungi	Rice, wheat	Dean et al. (2012)
*Puccinia* spp.	Fungi	Wheat, barley	Dean et al. (2012)
*Hyaloperonospora arabidopsidis*	Oomycete	Arabidopsis	Kamoun et al. (2015)
*Phytophthora infestans*	Oomycete	Potatoes, tomatoes	Kamoun et al. (2015)
*Phytophthora sojae*	Oomycete	Soybeans	Kamoun et al. (2015)
*Agrobacterium tumefaciens*	Bacteria	Grapes, walnuts	Mansfield et al. (2012)
*Pseudomonas syringae*	Bacteria	Tomatoes, beans	Mansfield et al. (2012)
*Xanthomonas oryzae*	Bacteria	Rice	Mansfield et al. (2012)
*Heterodera* spp.	Nematode	Soybeans, cereals	Jones et al. (2013)
*Meloidogyne* spp.	Nematode	Various crops (e.g., vegetables, cereals)	Jones et al. (2013)

#### 4.1.1 Bacterial pathogens

Bacterial phytopathogens represent a persistent threat to global agriculture, resulting in severe yield losses and economic setbacks. Historically, detection and identification hinged on labor-intensive, laboratory-based methods that were time-consuming and required technical expertise. Recent innovations, however, have ushered in a new era of handheld, easily deployable devices that deliver rapid, sensitive, and accurate on-site detection. Such tools empower farmers and agronomists to respond promptly, curbing the spread of bacterial diseases before they devastate crops.

For instance, the bacterial pathogen *Xylella fastidiosa*—a severe menace to Southern European olive groves—impedes xylem function, leading to plant wilting and eventual death. Handheld devices employing LAMP assays now enable in-field detection of *X. fastidiosa* DNA within half an hour. This accelerated process supports immediate decisions regarding containment strategies (Elbeaino et al., 2020). A similar approach addresses *Erwinia amylovora*, the causative agent of fire blight in apple and pear orchards. LFIAs facilitate real-time pathogen identification through a visible test line analogous to home pregnancy tests. With these simple, cost-effective assays, growers can swiftly engage in targeted pruning or other interventions, averting further spread (Braun-Kiewnick et al., 2011).

Microfluidic LOC platforms further streamline bacterial diagnostics. By integrating sample preparation, DNA amplification, and detection into a single, portable device, these systems yield results within an hour. A microfluidic platform developed for *Ralstonia solanacearum*, a bacterium responsible for wilt diseases in various crops, exemplifies this technology’s potential to guide proactive management decisions (Chu et al., 2024). Concurrently, the fusion of diagnostic tools with smartphone technology leverages built-in high-resolution cameras, computing power, and connectivity. A smartphone-based fluorescence *in situ* hybridization (FISH) method for *Pseudomonas syringae* allows real-time data capture and sharing, streamlining collaborative disease surveillance and rapid response strategies (Rodriguez-Moreno et al., 2008).

#### 4.1.2 Viral pathogens

Viral pathogens pose a formidable challenge to global crop production, frequently causing significant yield reduction and quality deterioration. Swift detection can prevent irreversible losses, yet traditional diagnostics like PCR-based methods require laboratory infrastructure, delaying timely interventions. The proliferation of portable, field-compatible assays has transformed this landscape by enabling immediate diagnosis and control measures at the outbreak’s origin.

For example, the Tomato Yellow Leaf Curl Virus (TYLCV), disseminated by the whitefly *Bemisia tabaci*, can result in catastrophic yield losses in tomato crops. Innovative, portable LAMP assays bypass the complexities of thermocycling and deliver accurate results in under 30 min, facilitating immediate removal of infected plants and vector management (Zhou et al., 2022). Similarly, Potato Virus Y (PVY) detection, crucial for maintaining seed quality and ensuring stable potato production, is now possible through LFIAs employing virus-specific antibodies. These user-friendly, on-site tests offer high sensitivity, specificity, and rapid results—ideal for non-specialist operators (Danks and Barker, 2000).

The integration of smartphone technology into viral diagnostics offers further versatility. High-resolution imaging combined with cloud-based data sharing and analytical applications streamlines surveillance efforts. A smartphone-adapted gold nanoparticle colorimetric assay for Cucumber Mosaic Virus (CMV) exemplifies how accessible, field-ready platforms can reliably detect viruses. Immediate data processing and geotagged information enhance disease mapping and resource allocation (Rafidah et al., 2016).

#### 4.1.3 Fungal pathogens

Fungal phytopathogens constitute another substantial impediment to global food production, notorious for persistent soilborne infections and rapid, widespread epidemics. Conventional laboratory-based fungal diagnostics are expensive, slow, and reliant on specialized expertise. The advent of portable detection devices has revolutionized how these threats are managed, ensuring swift identification and targeted countermeasures.

A prominent case is *Phytophthora infestans*, the pathogen behind late blight in potatoes and tomatoes—famously linked to the Irish Potato Famine. Using immunochromatographic LFIAs, farmers gain on-site confirmation of pathogen presence in minutes, expediting fungicide application or removal of infected material (Lu et al., 2020). Similarly, the soil-borne *Fusarium oxysporum*, capable of lingering in fields and causing devastating wilt diseases, can be detected through portable LAMP assays. These rapid tests enable growers to enact soil treatments promptly, adjust crop rotations, and mitigate subsequent losses (Katoh et al., 2021).

Microfluidic LOC systems further improve fungal pathogen diagnostics by streamlining sample preparation, nucleic acid amplification, and detection. Devices targeting *Sclerotinia sclerotiorum*, a widespread fungal pathogen affecting crops like soybeans and canola, provide quick and reliable confirmation of pathogen presence on the spot. Additionally, smartphone-coupled diagnostic tools, such as those for *Puccinia striiformis*, leverage portable microscopy attachments and image analysis software for immediate, in-field detection and communication of disease data (Zhan et al., 2014).

## 5 Performance, validation, and standardization

### 5.1 Field-applicable performance parameters

Field-based phytopathogen diagnostics provide essential advantages over traditional centralized laboratory testing, mainly by reducing reliance on labs, which often have long processing times and require specialized equipment and personnel. On-site diagnostic devices allow rapid pathogen detection at the infection site, enabling swift responses to prevent disease spreading. This capability is precious in agricultural regions with limited lab access, empowering farmers to make informed, timely decisions that positively impact crop health and yield.

Successful implementations of field diagnostics illustrate their effectiveness in managing crop diseases. For example, tomato crops face threats from pathogens like *Xylella fastidiosa* and TYLCV, which can cause severe yield losses if not caught early. Handheld LAMP-based assays for TYLCV have proven effective for rapid, accurate detection in the field, enabling farmers to quickly identify and remove infected plants, thereby reducing the spread of disease. In lettuce cultivation, lab-on-a-chip technologies have been instrumental in managing infections caused by *Sclerotinia sclerotiorum*, a pathogen that can devastate crops. These portable devices provide results within minutes, allowing targeted fungicide application to protect yields, a clear improvement over traditional lab methods that require days for results (Grabicoski et al., 2020).

Field diagnostics also significantly impact cereal crops, such as wheat, affected by rust caused by *Puccinia striiformis*. Wheat rust can substantially reduce yields if not promptly controlled. Smartphone-integrated devices allow farmers to detect rust early by capturing and analyzing images of infected leaves via an app, which can also share data with agricultural services for coordinated responses. Real-time analysis and data sharing enhance the effectiveness of interventions like fungicide application and crop rotation, ultimately safeguarding yields. Table 5 presents a performance comparison of various diagnostic devices for plant pathogen detection, detailing their target pathogen types, limits of detection (LOD), response times, and selectivity. This comparison highlights the strengths and applications of different technologies, ranging from rapid detection with high specificity to portable and cost-effective solutions for diverse pathogen types.

**Table 5:** Performance comparison of diagnostic devices for plant pathogen detection.

Device type	Pathogen type example	Limit of detection (LOD)	Response time	Selectivity	References
Electrolyte-gated organic transistor	Fungal pathogens (e.g., *Fusarium* spp.)	3 pM	Minutes	High selectivity for specific proteins	Parkula et al. (2020)
Nafion-glutamate oxidase biosensor	Bacterial pathogens (e.g., *Xanthomonas* spp.)	0.3 μM	Immediate	High against electroactive interferents	Pan and Arnold (1996)
BODIPY-based fluorescent probe	Fungal pathogens (e.g., *Botrytis* spp.)	0.12 nM	<1.5 s	High, selective for specific phytochemicals	Zhang et al. (2017)
Phage-based quartz biosensor	Bacterial pathogens (e.g., *Pseudomonas syringae*)	0.003 nM	~100 s	Excellent for enzyme-linked detection	Nanduri et al. (2007)
Integrated microfluidic NASBA	Viral pathogens (e.g., Tobacco Mosaic Virus)	Detects RNA from ~100 bacteria	<30 min	High pathogen-specific response	Dimov et al. (2008)
Nanodiagnostics	General plant pathogens (broad range)	Variable (depends on platform)	Rapid	Multiplexing with smart sensing features	Kashyap et al. (2016a)
Graphene FET biosensor	Fungal and bacterial pathogens	2 × 10^−18^ M	Real-time	High, effective even in ionic fluids	Sarker et al. (2023)
Lateral flow immunoassay (LFIA)	Viral and bacterial pathogens (e.g., PVY)	0.25 ng mL^−1^	5–20 min	Good selectivity via antibodies	Razo et al. (2018)
Surface plasmon resonance (SPR) biosensor	Bacteria	50 CFU mL^−1^	Minutes	High specificity via ligand-receptor binding	Wang et al. (2012)
Paper-based microfluidic device (μPAD)	Fungal, bacterial & viral pathogens	nM to pM range (depends on design)	<30 min	Moderate selectivity; portable & cost-effective	Martinez et al. (2010)
Quartz crystal microbalance (QCM) sensor	Bacteria	100 CFU/mL	Less than 10 min	High mass-sensitive detection	Ozalp et al. (2015)

### 5.2 Validations under resource-poor settings and impact assessment

Validation of phytopathogen diagnostic devices in resource-poor settings requires robust assessment of specificity, sensitivity, reproducibility, and fitness-for-purpose under field conditions. Resource limitations such as inadequate infrastructure and reagent stability issues necessitate adopting techniques like LAMP, which allows on-site detection without advanced equipment (De Jonghe et al., 2016). Validation begins with stakeholder input to identify pathogens and agroecological constraints, using local sample panels to ensure relevance. For example, validating portable diagnostic devices using local sample panels to detect *Ralstonia solanacearum* in Kenyan potato fields enhances their relevance and farmer confidence (Okiro et al., 2019). Comparative validation against “gold-standard” methods approved by organizations like IPPC or EPPO is crucial (Conraths and Schares, 2006). A study investigated a nested PCR method against gold-standard techniques, demonstrating its utility for detecting *Xanthomonas axonopodis* pv. *dieffenbachiae*, and emphasizing the role of EPPO-endorsed protocols (Chabirand et al., 2013).

A lateral flow device was developed, and PCR-based diagnostic methods were evaluated for *Xanthomonas campestris* pv. *musacearum* under field conditions in East Africa, showing high sensitivity and specificity for early disease detection (Hodgetts et al., 2015).

Ruggedness testing is equally vital, ensuring performance under harsh conditions such as fluctuating temperatures or dust. Devices like solar-powered portable PCR kits validated for maize lethal necrosis disease maintained reliability in extreme environments (Snodgrass et al., 2016). Usability testing ensures operational simplicity for users with minimal training, as demonstrated by lateral flow devices (LFDs) for plant pathogen detection, with local technicians in field conditions achieving reliable results through simple operational protocols (Danks and Barker, 2000).

Periodic reassessment of diagnostic tools is crucial to capture pathogen evolution and maintain their robustness. Adaptive frameworks with iterative calibration and field survey feedback ensure diagnostics remain relevant. For example, integrating evolutionary dynamics with immune responses provides insights into pathogen adaptation and highlights iterative calibration’s role in robust management (Restif and Grenfell, 2006). Advances like adaptive sequencing enhance real-time detection and resistance profiling, demonstrating the significance of continuously updated methods (Cheng et al., 2022). Together, these approaches emphasize dynamic, flexible systems essential for addressing evolving pathogen diagnostics and management challenges.

For instance, validated kits for Cassava Brown Streak Virus in Eastern Africa reduced pesticide sprays, improving profitability and reducing environmental harm (Patil et al., 2015). Scalability and sustainability are assessed by evaluating whether diagnostics can be produced locally or maintained with minimal external inputs. Gender-inclusive approaches ensure equitable benefits, particularly for women who play a significant agricultural role (Jost et al., 2015). Integrated validation frameworks that combine diagnostics with data analytics show promise. Handheld LAMP devices paired with smartphone imaging provide real-time geo-referenced disease data for community-wide surveillance, enhancing decision-making and agricultural resilience (Paul et al., 2021). Validated diagnostics in resource-poor settings catalyze sustainable agrarian intensification, resilience to emerging pests, and improved livelihoods.

Furthermore, portable diagnostic devices contribute to environmental sustainability by supporting precision agriculture practices. Accurate, on-site detection allows for targeted treatment of affected plants, reducing the reliance on broad-spectrum pesticide applications. This precision minimizes unnecessary chemical usage, thereby lessening the environmental impact of agricultural activities. Potable diagnostics are vital in advancing sustainable farming methodologies by promoting efficient use of resources and decreasing the potential for harmful side effects on ecosystems. Table 6 compares the three most commonly used portable phytopathogen diagnostic technologies based on their attributes.

**Table 6:** Comparison of portable diagnostic technologies based on attributes.

Attribute	Detection sensitivity	Sample processing time	Cost	Field applicability	References
Handheld analyzers	Moderate to high; varies by pathogen type	Fast (10–30 min)	Moderate; often higher for advanced models	High; portable and user-friendly	Crocombe (2018)
Smartphone-integrated tools	Moderate; can be enhanced with additional sensors	Variable (5–45 min)	Low to Moderate; dependent on smartphone compatibility	Very high; accessible and widely usable	Zhang and Liu (2016)
Lab-on-a-chip systems	High; suitable for multiplexed detection	Fast (5–20 min)	Higher initial setup cost; low per-test cost	Moderate; requires specific operating conditions	Zhu et al. (2020)

### 5.3 Regulatory, quality assurance, and standardization challenges

While offering rapid, sensitive, and specific diagnostics, portable diagnostic tools for detecting phytopathogens face significant hurdles that limit their effectiveness and adoption. Despite advancements like LFIA and LAMP assays, these devices struggle with sensitivity and specificity under environmental stressors such as temperature and humidity, leading to false positives and negatives. Detecting pathogens at low concentrations remains challenging, hindering their application in early disease detection. Overcoming these technical limitations through enhanced sensitivity and specificity across diverse environments is crucial (Ali et al., 2021; Dey et al., 2023; Archana et al., 2024).

Standardization and quality assurance are equally critical for ensuring the reliability of these tools. A lack of universal testing protocols and calibration procedures undermines result consistency, complicating regulatory approvals and limiting scalability. This inconsistency reduces user confidence, especially among smallholder farmers and agricultural workers, who rely on these devices for on-site diagnostics (Ali et al., 2021). Advances in nanotechnology, molecular diagnostics, and IoT-enabled systems have begun addressing these gaps. For instance, nanotechnology enhances sensitivity and specificity, enabling reliable on-site detection via accessible devices such as smartphones (Li Z. et al., 2020). Molecular diagnostics like real-time PCR and microchip PCR systems integrate built-in quality assurance features such as temperature regulation and amplification monitoring, ensuring dependable pathogen detection in diverse settings (Koo et al., 2013). IoT-enabled tools provide real-time data collection and cloud-based quality assurance, promoting consistent accuracy in field conditions (Buja et al., 2021).

Durability and operational challenges further constrain portable device usability. Harsh field conditions degrade performance, and maintaining devices, including calibration and component replacement, is often infeasible in remote areas. Standardized molecular protocols, like isothermal amplification and next-generation sequencing, reduce variability through uniform sample preparation and device calibration, while user-friendly tools such as biosensors and lateral flow assays improve accessibility for growers and field workers without requiring extensive training (Hariharan and Prasannath, 2021).

Economic barriers compound these challenges, with high upfront costs, recurring consumable expenses, and training requirements making these devices less accessible to smallholder farmers. Inadequate training exacerbates the risk of data misinterpretation, potentially worsening pathogen spread. Additionally, regulatory and ethical considerations, including compliance with regional agricultural laws and data privacy issues linked to cloud-based and smartphone technologies, create further obstacles to widespread adoption. Table 7 illustrates the advantages and limitations of various portable phytopathogen diagnostic devices.

**Table 7:** Advantages and limitations of portable diagnostic devices.

Attribute	Advantages	Limitations	References
Rapid results	Provides quick results, often within minutes, aiding timely intervention	Some devices may sacrifice accuracy for speed, leading to potential false results	Buja et al. (2021)
Cost-effectiveness	Reduces costs by eliminating the need for laboratory facilities and specialized equipment	Initial costs for advanced portable devices can be high, limiting accessibility	Hohenstein et al. (2017)
Portability	Highly portable, enabling on-site diagnostics directly in the field	Portability may be compromised in devices requiring external power sources	Wu et al. (2021)

### 5.4 Commercial relevance of portable phytopathogen diagnostic tools

Portable phytopathogen diagnostic tools hold immense commercial potential by mitigating economic losses, enhancing market stability, and driving innovation in agriculture. Early detection of plant diseases minimizes yield losses, stabilizes market supplies, and ensures product quality, addressing challenges from asymptomatic infections and emerging diseases. These tools streamline field applications by eliminating the reliance on centralized laboratories, with real-time PCR systems and nanotechnology-based devices offering rapid and sensitive detection. Their affordability, particularly with smartphone-integrated systems, makes them viable for resource-limited settings, fostering inclusivity in agricultural markets (Paul et al., 2021).

Integration with IoT amplifies their value by linking diagnostics to real-time data management systems, improving disease monitoring and decision-making. Additionally, their applicability extends beyond agriculture to industries like water quality testing, food safety, and environmental monitoring, enabling manufacturers to diversify revenue streams (Islam et al., 2023). These tools also support sustainable agriculture by reducing agrochemical use through precise interventions and meeting consumer demand for eco-friendly, residue-free produce.

## 6 Challenges, limitations and future innovations

### 6.1 Technical limitations

Current portable phytopathogen diagnostic devices face significant technical limitations despite miniaturization and field usability advances. A key constraint is throughput; while laboratory methods like high-throughput qPCR or NGS can process large sample volumes efficiently, portable devices are restricted to a few targets per run, limiting their utility for large-scale surveillance. Handheld molecular assays like LAMP improve speed and convenience but are constrained by their inability to handle large sample loads in field conditions, as seen in their limited use for detecting pathogens like *Phytophthora infestans* and *Botrytis cinerea* in agricultural settings (Ristaino et al., 2020).

Multiplexing remains another significant hurdle. Detecting multiple pathogens simultaneously is essential for cost-effectiveness but is limited by constraints such as the cross-reactivity of primers and uneven assay performance. Attempts at multiplexing for pathogens like *Pseudomonas syringae*, *Xanthomonas campestris*, and *Ralstonia solanacearum* often result in reduced accuracy, while detecting phylogenetically diverse pathogens (bacteria, fungi, oomycetes, viruses) poses additional biochemical and logistical challenges (Umesha and Avinash, 2015).

Stability of reagents and devices is critical for field deployment. Reagents must withstand environmental fluctuations, but real-world use often shows degradation in sensitivity, as seen in LAMP-based assays for plant viruses requiring cold-chain logistics. Immunoassays also face stability issues, with antibodies losing efficacy under field conditions (Thekisoe et al., 2009).

In West Africa, molecular assays for detecting cassava pathogens like CBSV struggled with reagent stability under high temperatures (Abarshi et al., 2012). Similarly, vineyard surveillance for pathogens like *Plasmopara viticola* and *Erysiphe necator* revealed inefficiencies in multiplexed assays and difficulties in managing plant inhibitors in field settings (Possamai and Wiedemann-Merdinoglu, 2022).

Engineering refinements in microfluidics, robust reagent formulations, advanced bioinformatics tools, and integrated digital platforms are essential to address these issues. Emerging methods like CRISPR-based diagnostics show promise but require further validation. Collaboration across disciplines, investment in infrastructure, and regulatory harmonization are necessary to bridge the gap between laboratory capabilities and field requirements, paving the way for more effective integrated pest management systems.

### 6.2 Advances and emerging technologies

Traditional diagnostic methods, though reliable, are impractical for on-site use, prompting the development of portable systems that integrate molecular biology, engineering, and data analytics. Advances in isothermal amplification techniques like LAMP and recombinase polymerase amplification (RPA) have enabled quick and efficient pathogen detection without requiring complex thermal cycling equipment. These methods have been successfully applied to detect pathogens such as *Phytophthora sojae* in soybean fields and *Ralstonia solanacearum* in bacterial wilt cases, providing actionable insights for immediate field interventions (Miles et al., 2015).

CRISPR-Cas systems have further revolutionized diagnostics, offering unparalleled specificity through programmable Cas12 and Cas13 proteins, enabling rapid pathogen identification from simple samples. Such technologies are adaptable and promise applications for various plant diseases (Li et al., 2022; Prasad et al., 2022). Rapid and efficient insulated isothermal PCR method for detecting *Fusarium oxysporum* directly from banana crop samples, supporting early diagnosis and containment efforts (Chang et al., 2022).

Emerging technologies such as nanopore sequencing are also transforming field diagnostics. Handheld devices like the Oxford Nanopore MinION deliver real-time pathogen identification, offering strain-level precision even in remote locations, although they currently require technical expertise (Player et al., 2020). Smartphones have further enhanced accessibility by integrating high-resolution imaging and cloud-based data sharing for real-time pathogen detection and geotagged disease monitoring. Smartphone-based fluorescence detectors exemplify the potential for user-friendly diagnostic tools that empower farmers with timely insights (Zhang et al., 2023).

The future of portable diagnostics lies in integrating multiplexed detection, machine learning, and user-friendly interfaces to democratize agricultural disease management. Innovations in synthetic biology, biosensors, and nanotechnology are expected to enhance diagnostic accuracy and accessibility further, empowering farmers to mitigate threats with minimal delay and ensuring sustainable agricultural practices in a changing climate. Figure 15 visualizes various phytodiagnostic techniques used in agriculture.

### 6.3 Scalable manufacturing, cost-reduction strategies, and supply chain considerations

Scalable manufacturing and cost-reduction strategies for portable phytopathogen detection devices focus on optimizing materials, modular designs, and streamlined production processes. Paper-based lateral flow assays are cost-effective due to their simplicity, compatibility with point-of-need applications, and reliance on mass-produced cellulose membranes (Sena-Torralba et al., 2022). Polymers like cyclic olefin copolymer (COC) and poly(methyl methacrylate) (PMMA), used in microfluidic chip fabrication, offer scalable manufacturing via injection molding, while low-cost sensors, such as LEDs and smartphone cameras, reduce reliance on specialized hardware (Liu et al., 2024). Modular designs enable standardization of components, such as interchangeable reagent cartridges, enhancing scalability and reducing per-unit costs.

Reagent stabilization and simplified sample preparation are critical for scalability. Lyophilized primers and isothermal amplification methods like LAMP and RPA enable ambient storage and reduce cold-chain dependence (Carter et al., 2017). Integrated sample-to-answer systems employing miniaturized nucleic acid extraction modules have been successfully used to detect pathogens like *Phytophthora infestans* in potato fields, empowering smallholder farmers with rapid diagnostic capabilities (Paul et al., 2021). Bulk purchasing of reagents, automated assembly lines, and robotic manufacturing streamline production while maintaining reliability (Ortiz et al., 2017).

Collaborative approaches play a vital role in cost reduction. Partnerships between public and private sectors, supported by agencies like the International Plant Protection Convention (IPPC), facilitate technology transfer, standardization, and large-scale production (Boyer et al., 2016). Domestic manufacturing reduces import costs and enhances local availability in low- and middle-income countries, while philanthropic and governmental support ensures affordability. Automated quality control measures using standardized reference strains, assuring product reliability across regions (Adiga et al., 2018).

Real-world examples highlight these strategies. Mass-produced PCR-based lateral flow kits for Cassava Brown Streak Virus in East Africa demonstrated reduced costs through local manufacturing and smartphone-based detection (Munguti et al., 2024). Similarly, a low-cost lateral flow device for field detection of *Xanthomonas campestris* pv. *musacearum* in banana plantations aids in rapid outbreak mitigation and reduces losses (Hodgetts et al., 2015). Such efforts create a sustainable ecosystem for innovation, enabling widespread deployment of portable phytopathogen detection devices and bolstering global food security.

### 6.4 Linking diagnostics to global surveillance and early warning system

Linking portable phytopathogen diagnostics to global surveillance and early warning systems integrates advanced molecular detection, sensor technologies, and data-driven networks for rapid on-site disease assessment. Portable tools, such as isothermal amplification techniques like LAMP, enable quick and reliable detection without laboratory infrastructure, producing results within 30–60 min (Parida et al., 2008). CRISPR-based detection platforms add specificity and sensitivity, making pathogen identification highly precise and accessible. Smartphone imaging and biosensor systems enhance portability by integrating high-resolution cameras, image recognition, and cloud-based analysis, enabling real-time data sharing with global networks. These advancements align with global initiatives like the IPPC and FAO’s surveillance frameworks, ensuring that diagnostic data feed into platforms like the International Phytosanitary Portal and GPPIS for immediate access by plant health authorities (International Plant Protection Convention (IPPC), 2016). For example, LAMP kits reduce instances of *Phytophthora* spp. and *Phytophthora cactorum* in strawberry fields (Siegieda et al., 2021) and rapid diagnostics curtailing downy mildew in vineyards through timely fungicide application (Radhey et al., 2024). As climate shifts alter pathogen distributions, portable diagnostics ensure real-time disease mapping and swift interventions, fortifying global capacity to mitigate threats (Buja et al., 2021).

Empowering local networks through training in assay interpretation, data logging, and sample handling builds decentralized surveillance systems capable of early pathogen detection. Public-private partnerships can improve kit affordability and innovation, while international agencies ensure cross-regional data accuracy through proficiency testing (Markell et al., 2020). Emerging technologies, such as drones and satellites equipped with diagnostic tools, offer the potential for real-time infection mapping and outbreak prediction through integrated data systems. Such innovations advance global preparedness, fostering resilient and collaborative responses to safeguard food security and biodiversity (Abbas et al., 2023).

### 6.5 Policy and stakeholder engagement

The development of portable phytopathogen detection devices offers significant potential for modern agriculture by enabling rapid and accurate disease diagnosis to reduce crop losses. To ensure their successful adoption, robust policies, and stakeholder engagement are crucial. Governments should incentivize research and development through grants and tax benefits while fostering public-private partnerships to accelerate prototyping and testing. Clear regulatory guidelines are essential to guarantee safety, reliability, and data security, while privacy policies should balance farmer confidentiality with the benefits of aggregated data for disease monitoring and early warning systems. Market accessibility can be enhanced by subsidies, reduced tariffs, and microcredit systems, particularly for smallholder farmers in low-income regions.

Active stakeholder involvement is critical throughout the development and deployment processes. Farmers, as primary users, must be included in co-design efforts to ensure the devices meet real-world needs. Research institutions and agricultural organizations are instrumental in tailoring detection mechanisms to region-specific pathogens and validating device performance. Collaboration with agrarian technology companies supports mass production and ensures alignment with market demands, while engagement with policymakers facilitates streamlined regulatory approvals and integration with agricultural health priorities. International organizations like the FAO can provide technical assistance, advocate for standardization, and support deployment in underserved areas. Additionally, NGOs can enhance access in remote farming communities.

Building capacity among agricultural extension workers ensures that the devices are correctly utilized, maximizing their effectiveness. Cost barriers and adoption resistance can be addressed through subsidized manufacturing, NGO partnerships, training programs, and demonstration projects to build trust and demonstrate value. Interoperability issues can be resolved with open data standards and modular designs, enhancing device usability across different systems and regions. A cohesive approach integrating strong policies with comprehensive stakeholder engagement is vital for the widespread adoption of these diagnostic technologies. Such efforts will strengthen agricultural productivity and resilience, ensuring these innovations fulfill their potential to combat crop diseases and enhance global food security.

## 7 Conclusion

Portable diagnostic technologies for plant pathogens present a transformative opportunity in agriculture by enabling rapid, on-site detection that bridges the gap between disease identification and response. These devices offer significant benefits, particularly in their capacity to reduce dependency on centralized laboratory facilities, thus democratizing access to timely and precise diagnostic tools. Current advancements have integrated smartphone technologies, IoT, and machine learning, underscoring the potential of portable diagnostics to become indispensable tools in modern agricultural practices. By fostering rapid pathogen detection, these technologies directly support sustainable agriculture, enhancing food security through improved plant health management. Portable diagnostic tools contribute to reduced chemical usage and allow for more precise interventions, thereby minimizing environmental impacts and supporting long-term ecological balance.

For stakeholders, including researchers, policymakers, and industry players, the potential of portable diagnostics in agriculture calls for coordinated efforts to address existing challenges such as standardization, cost barriers, and regulatory considerations. Researchers should focus on improving the sensitivity and specificity of these devices and on developing robust, field-deployable technologies that can withstand diverse agricultural conditions. Policymakers play a crucial role in supporting frameworks that encourage the adoption of portable diagnostic tools while maintaining data privacy and security standards. Meanwhile, industry players are encouraged to invest in and develop cost-effective, scalable solutions accessible to small-scale farmers and large agricultural operations.

Looking ahead, the future of portable diagnostics in agriculture is promising. Emerging technologies, such as nanotechnology and microfluidic systems, will enhance these devices’ precision and utility, allowing for early detection of pathogens even at low concentrations. As agriculture increasingly embraces digital transformation, integrating portable diagnostics with predictive analytics, precision farming, and real-time data-sharing platforms could revolutionize how plant diseases are managed. This shift towards a more connected, data-driven approach holds the potential to mitigate crop losses and bolster global food security by enabling proactive, tailored disease management solutions across diverse agricultural landscapes.
